# Activation and maturation of antigen-specific B cells in nonectopic lung infiltrates are independent of germinal center reactions in the draining lymph node

**DOI:** 10.1038/s41423-025-01285-8

**Published:** 2025-04-11

**Authors:** Sarah-Sophie Schacht, Josefine Graffunder, Pawel Durek, Jonas Wehrenberg, Annette Siracusa, Charlotte Biese, Mir-Farzin Mashreghi, Kevin Thurley, Laura Bauer, Andreas Hutloff

**Affiliations:** 1 Institute of Immunology, University Hospital Schleswig-Holstein, Kiel, Germany; 2 Chronic Immune Reactions, German Rheumatism Research Centre, A Leibniz Institute, Berlin, Germany; 3 Therapeutic Gene Regulation, German Rheumatism Research Centre, A Leibniz Institute, Berlin, Germany; 4 German Center for Child and Adolescent Health (DZKJ), partner site Berlin, Berlin, Germany; 5 Institute for Experimental Oncology, Biomathematics Division, University Hospital Bonn, Bonn, Germany; 6 Institute of Clinical Molecular Biology, University Hospital Schleswig-Holstein, Kiel, Germany

**Keywords:** Lung inflammation, germinal center, somatic hypermutation, peripheral T helper cells, macrophages, immunoglobulin A, Cellular immunity, Humoral immunity

## Abstract

Pulmonary T and B cells are important for protection of this mucosal barrier site. While viral infections lead to the development of ectopic lymphoid structures highly similar to those in germinal centers in secondary lymphoid organs, little is known about how T/B cooperation occurs in the unstructured, diffuse tissue infiltrates characteristic of autoimmune diseases and nonviral infections. Using a mouse model of interstitial lung inflammation, we found that naive B cells are directly activated in lung tissue. Despite the absence of any germinal center-like structures, the interaction of B cells with peripheral T helper cells results in efficient somatic hypermutation and class switching. As antigen-presenting cells, macrophages are critical for this process. Unique B-cell repertoires indicated that the lung response was autonomous from the lung-draining lymph node. Only lung GC-like B cells were switched to IgA and had a broader repertoire, making them ideal candidates for producing broadly neutralizing immunoglobulins against respiratory pathogens.

## Introduction

The lung represents one of the largest mucosal surfaces in the body and is constantly exposed to the environment via the airways. It is an entry site for various airborne pathogens, such as influenza, SARS-CoV-2 or *Mycobacterium tuberculosis*, and is therefore an immunologically highly relevant barrier tissue [1, 2]. In addition to innate immune cells, pulmonary T and B cells contribute significantly to local protection against pathogens. On the other hand, T and B cells in the lung are involved in the development of chronic inflammatory interstitial lung diseases [3]. The constant low level of inflammation in the lung may promote the selection of autoreactive T and B cells, turning the lung into a priming site for systemic autoimmune diseases such as rheumatoid arthritis [4]. However, apart from influenza A infection models, the role of T and B cells in the lung is poorly understood.

The activation and B cell receptor (BCR) affinity maturation of B cells typically take place in secondary lymphoid organs (SLOs), where the specialized population of T follicular helper (Tfh) cells drives B cell selection during the germinal center (GC) response [5]. The complex microstructure of SLOs with separate T and B cell zones as well as follicular dendritic cells (FDCs), a stromal cell population specialized in the long-term retention of antigens, provides optimal conditions for the selection of high-affinity antibody-producing plasma cells and the generation of memory B cells. Under chronic inflammatory conditions, so-called ectopic lymphoid structures (ELSs), also known as inducible bronchus-associated lymphoid tissue (iBALT) in the lung, can develop in nonlymphoid tissues [6]. Structurally and functionally, ELSs fully resemble SLOs, including the presence of FDCs, GC B cells, and Tfh cells, which are also known as T resident helper (Trh) cells in the lung [7, 8]. The development of ELSs requires rather strong immunological stimuli, such as a viral infection [9]. In line with that, other pulmonary pathogens, such as *Streptococcus pneumoniae* [10] and *Pseudomonas aeruginosa* [11], or chronic inflammatory lung diseases, such as sarcoidosis [12], do not lead to the development of iBALT but rather cause the development of unstructured infiltrates of T and B cells in the lung.

In this context, the recently discovered population of peripheral T helper (Tph) cells is of central interest [9]. Tph cells are specialized to reactivate B cells in inflamed nonlymphoid tissues in the absence of ELS. They lack expression of the classical Tfh cell markers CXCR5 and Bcl-6 but nevertheless can provide efficient local help for B cells and their differentiation into plasmablasts owing to high expression of CD40L and IL-21 [13]. Tph cells are recognized as a potential disease-driving population in many chronic inflammatory diseases [14]. Nevertheless, it is currently unclear whether Tph cells are capable of activating and differentiating naive B cells in the absence of the complex microstructure of an SLO [14, 15].

Here, to characterize antigen-specific B cells in inflamed nonlymphoid tissue, we leveraged a mouse model of lung inflammation that allows the simultaneous analysis of antigen-specific T and B cells in the lung-draining lymph node and in the lung. Specifically, we investigated how B cell activation, differentiation, and affinity maturation can take place in the absence of the ordered structure of the GC. These questions are highly clinically relevant, both in terms of preventing the generation of autoreactive B cells in the context of chronic inflammation and in terms of promoting the development of tissue-resident memory B cells in barrier organs for vaccination purposes.

## Results

### B cells in the lung develop a GC-like phenotype despite the absence of any GC-specific structures

**Fig. 1:**
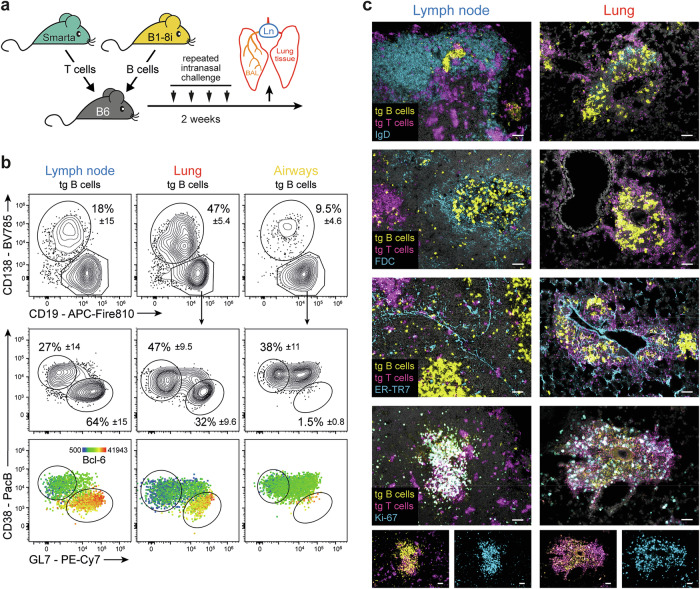
Lung-infiltrating B cells develop a GC-like phenotype despite the absence of iBALT structures. **A** Mouse model for the analysis of antigen-specific T and B cells in the lung and lung-draining lymph nodes. T cells from Smarta T-cell receptor transgenic mice (recognizing LCMV GP1 [43]) and B cells from B1-8i BCR knock-in mice (recognizing nitrophenol (NP) [45]) are adoptively transferred into C57BL/6 mice. Recipients are then immunized repeatedly with a cognate antigen (Smarta peptide and NIP coupled to mouse serum albumin as a nonimmunogenic carrier) and LPS as an adjuvant. After development of full lung inflammation, antigen-specific T and B cells (identified by the congenic markers Thy-1.1 and CD45.1, Supplementary Fig. S1) from lung tissue, airways (bronchoalveolar lavage (BAL)) and the lung-draining lymph node (Ln) can be analyzed by flow cytometry or immunohistology. **B** Phenotypes of antigen-specific B cells from lymph nodes, lung tissue, and airways analyzed on day 17. Flow cytometry staining for plasmablasts (CD19^low^ CD138^+^) and GC-like B cells (CD19^+^ CD38^low^ GL7^+^ Bcl-6^+^). Plots are concatenated from a representative experiment (out of more than 20 experiments) with six mice; percentages represent the means ± SDs. The rainbow scale in the lower panel indicates the expression (geometric mean) of Bcl-6. **C** Immunohistology analysis of antigen-specific T and B cells and nuclei (DAPI, gray) in combination with IgD, FDC (CD21/35^+^), stromal cells (ER-TR7^+^), or the nuclear proliferation antigen Ki-67. The scale bars represent 50 µm. Representative data from two independent experiments with a total of 15 mice are shown

We employed an established transgenic mouse model of lung inflammation (Fig. 1A) [16] to analyze antigen-specific T and B cells in inflamed lung tissue, airways, and lung-draining lymph nodes. Plasmablasts (CD19^low^ CD138^+^) were present at all three sites, whereas B cells with a GC-like phenotype (CD38^low^ GL7^+^ Bcl-6^+^) were found in the lymph nodes and lungs but not in the airways (Fig. 1B). Notably, iBALT structures do not develop in this protein-based immunization model [13], and T and B cell infiltrates in the lung lack any structural organization, such as separate T and B cell zones (Fig. 1C). Furthermore, FDCs, which are a hallmark of GC structures, were absent in the lung, although other stromal cell populations (ER-TR7^+^) were identified, and T and B cells were highly activated (Ki-67^+^) in both organs. B-cell activation and differentiation in this mouse model are strictly dependent on cognate antigen and T-cell help (Supplementary Fig. S2A). As previously reported [13], lung-infiltrating T cells exhibited a Tph phenotype lacking expression of CXCR5, whereas T cells in the lymph node presented a Tfh cell phenotype with coexpression of CXCR5 and PD-1 (Supplementary Fig. S2B). IL-21 production was similar across both cell types shortly after activation but declined more rapidly in the lungs than in the lymph nodes. Notably, lung infiltrating T and B cells remained within small infiltrates even after complete resolution of inflammation [13]. Three months after the last antigen application, B cells in the lungs and lung-draining lymph nodes exhibited a resting memory phenotype ([13] and Supplementary Fig. S2C). However, upon reactivation, they rapidly differentiated into GC (-like) B cells and plasmablasts (Supplementary Fig. S2C).

**Fig. 2:**
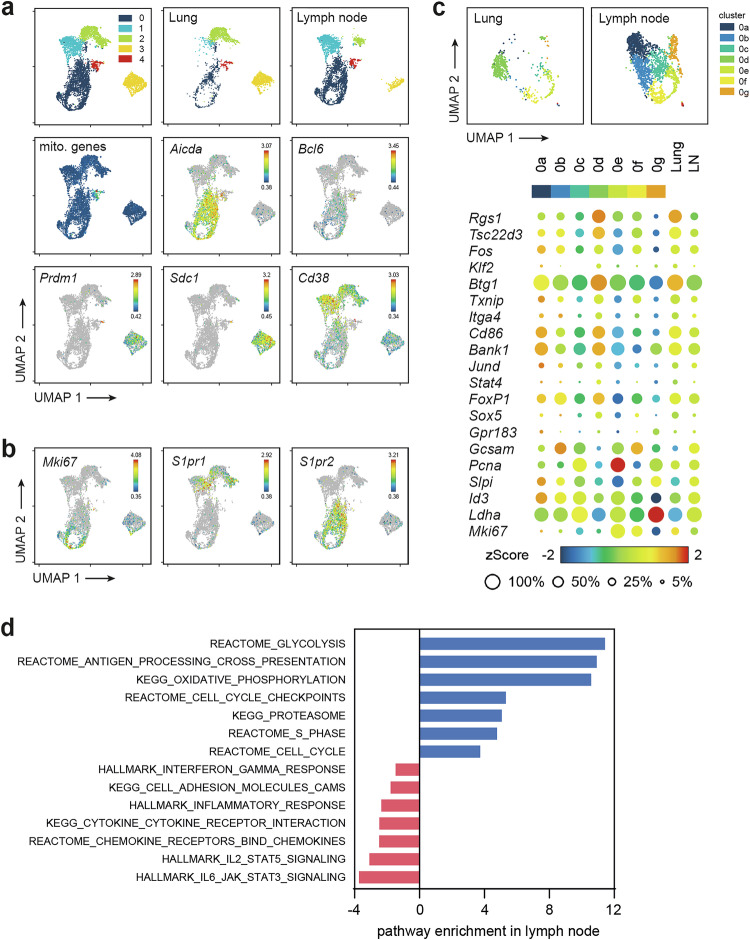
A single-cell transcriptome identifies distinct B cell subpopulations in lung and lymph node. Antigen-specific B cells were isolated from the lungs and lung-draining lymph nodes on day 17 and further processed for single-cell RNA sequencing. **A** Combined UMAP representation of lymph node- and lung-derived B cells. The expression of Aicda, which encodes activation-induced cytidine deaminase (AID), and the GC B-cell-specific transcription factor Bcl6 identified cluster 0 as GC-like B cells. Cluster 3 represents plasmablasts, as these cells all expressed high levels of Prdm1 (encoding Blimp-1) and Sdc1 (encoding CD138). Clusters 1 and 2 corresponded to the CD38^high^ population observed via flow cytometry. The cells in cluster 4 contained a high percentage of mitochondrial genes and were therefore considered dead or low-quality cells and excluded from further analysis. The expression levels of the indicated genes are shown on a rainbow scale. **B** UMAP representation of the typical GC B-cell-associated markers Mki67 (encoding Ki-67), sphingosine-1-phosphate receptor 1 (S1pr1; low in GC B cells), and S1pr2. **C** UMAP subclustering of GC-like cells (cluster 0 in panel a) and bubble plots showing the expression of the top DEGs between lung and lymph nodes with known functions for B cells. The color scale shows the z scores of the average expression of a gene within the indicated cluster. Bubble sizes correspond to the fraction of cells expressing a particular gene within the indicated cluster. **D** Selected pathways differentially expressed (p_adj_ < 0.05) between lung and lymph node GC-like B cells. The data are representative of two independent experiments: one experiment with total antigen-specific B cells from the lung and lymph nodes pooled from 10 animals (2565 lung and 3788 lymph node cells) and one experiment with sorted GC-like B cells from a single mouse (655 lung and 2176 lymph node GC cells)

Although lung-infiltrating B cells exhibit a GC-like phenotype on the basis of the markers CD38, GL7 and Bcl-6, they are generated in a highly different microenvironment than lymph nodes. Hence, to study the phenotypic differences in more detail, we sorted antigen-specific B cells from the lymph nodes and lungs for single-cell RNA sequencing (Fig. 2A, B). Uniform manifold approximation and projection (UMAP) of their transcriptomes revealed the same major subpopulations as those identified via flow cytometry, namely, GC (-like) B cells (cluster 0), CD38^high^ B cells (clusters 1 and 2), and plasmablasts (cluster 3) (Fig. 2A). For a more detailed analysis, we subclustered all cells from GC-like cluster 0 into eight new clusters (see Supplementary Fig. S3A for the top 5 genes defining each subcluster). Clusters 0a, 0b and 0c were specific for lymph node cells, whereas cluster 0 d was lung specific (Fig. 2C). A total of 467 genes were differentially expressed between lymph node and lung GC-like B cells (p_adj_ < 0.05 and log2-fold change > 1.2). The bubble plots show a selection of the top regulated genes with known functions in B cells. Global pathway analysis revealed that genes regulating the cell cycle, proliferation and metabolism were highly expressed in lymph node-derived GC B cells, whereas chemokine pathways were strongly represented in lung-derived B cells (Fig. 2D and Supplementary Fig. S4). Somewhat surprisingly, genes of the AID complex and genes involved in DNA repair, indicative of somatic hypermutation (SHM) and class switch recombination as two central events in the GC reaction, did not show major differences between lung- and lymph node-derived B cells (Supplementary Fig. S3B).

Taken together, these findings indicate that the gene expression changes in GC-like B cells from the lung and lymph nodes slightly differed at the transcriptional level, but these gene expression changes did not indicate any altered biological function.

### B cells are directly activated in the lung

**Fig. 3:**
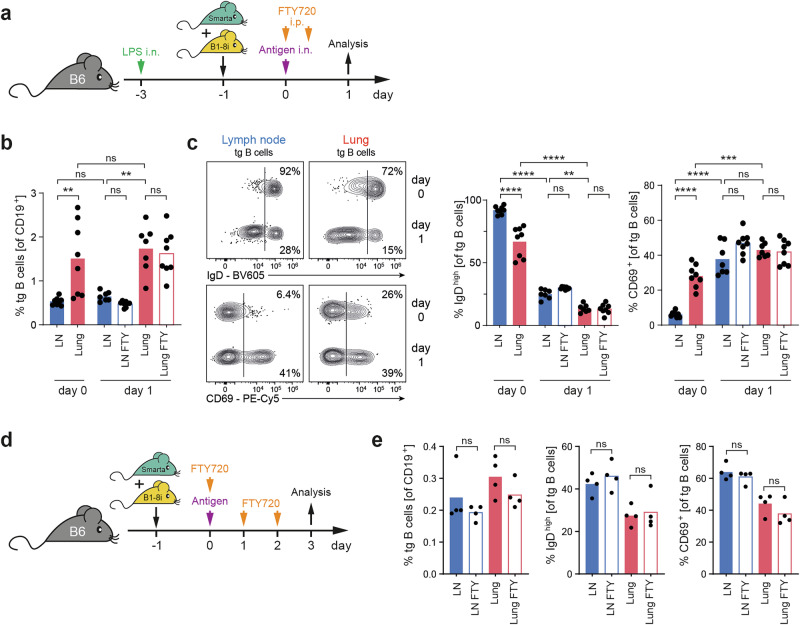
Naive B cells can be activated directly in lung tissue. **A** Experimental setup for the analysis of early B-cell activation in the lung. The mice received a single dose of 5 µg of LPS intranasally, which increased the frequency of endogenous B cells in the lung from approximately 0.8 to 2.2%. Three days later, antigen-specific T and B cells (the latter sorted for CD62L^high^ cells) were adoptively transferred, and the mice were immunized i.n. with antigen and LPS as adjuvants the next day. One group was injected with FTY720 i.p. at the time of antigen application and 12 h later. **B** Antigen-specific B cells (shown as the frequency of total B cells) in lung and lung-draining lymph nodes on day 0 and day 1, with and without FTY720 (FTY) treatment. **C** Expression of IgD and CD69 on antigen-specific B cells on day 0 and day 1. Vascular lymphocytes were excluded from analysis by i.v. injection of antibodies against CD3 and B220 immediately before sacrifice. Flow cytometry plots display one representative animal. **D** For analysis on day 3, the LPS pretreatment was omitted. One group received FTY720 at the time of antigen application and 24 and 48 h later. **E** Frequencies of antigen-specific B cells and the expression of IgD and CD69. The bar graphs show pooled data from two independent experiments with seven/eight animals per group **B, C** or from one representative experiment out of two **E**. Each animal is depicted by a dot, and the bar graphs indicate the means. ns, *p* ≥ 0.05; **, *p* < 0.01; ***, *p* < 0.001; ****, *p* < 0.0001

In principle, the high similarity between GC-like B cells in lymph node and lung tissue could be the result of a continuous exchange of cells between both organs. It is generally believed that the activation of naive B cells can only take place in SLO or at least requires the presence of ectopic lymphoid structures. However, naive T and B cells not only constantly recirculate through the SLO but also through nonlymphoid tissues [17, 18]. Under steady-state conditions, between 0.5 and 5% naive B cells were found in the lung tissue, depending on the age and housing conditions of the mice. Young SPF mice kept in individually ventilated cages (IVCs) have little exposure to environmental antigens and very few B cells in their lungs, making analysis of the even smaller population of antigen-specific B cells difficult. Therefore, we promoted B-cell recruitment to the lung by inducing sterile inflammation with low-level LPS. Three days later, antigen-specific B cells from B1-8i mice were adoptively transferred (Fig. 3A). These transferred cells were distributed throughout the whole body, resulting in a frequency of approximately 0.5% antigen-specific B cells among all CD19^+^ B cells in the lymph node and as high as 1.5% in the lung (Fig. 3B). Antigen-specific B cells do not divide within the first 24 hours, and indeed, their frequency did not increase on day 1 after stimulation, while we observed downregulation of IgD and upregulation of CD69 both in lymph node and lung tissue (Fig. 3C). Of note, a substantial proportion of B cells in the lung already express CD69 without any antigen stimulation, suggesting a tissue-induced upregulation of CD69 in an otherwise naive phenotype as previously described for T cells [17]. Finally, to formally exclude B-cell migration as a major determinant of humoral immunity in the lung, the animals were treated with the sphingosin-1-phosphate receptor antagonist fingolimod (FTY720), which blocks lymphocyte egress from the lymph node. This treatment neither altered the frequency of transgenic B cells in the lungs nor their early activation (Fig. 3B, C), confirming that B-cell activation in the lungs is independent of their activation in the lung-draining lymph nodes. Similar results were obtained in an experiment analyzed on day three. Here, the absence of LPS pretreatment revealed that B-cell activation is independent of the induction of sterile lung inflammation (Fig. 3D, E).

Overall, the unchanged frequency of transgenic B cells in the lung between day 0 and day 1, combined with unaltered B-cell frequencies in the presence of a sphingosin-1-phosphate receptor antagonist, argues against substantial migration of B cells from the lymph nodes to the lungs at early time points.

### Macrophages constitute the major antigen-presenting cell population in the lung

**Fig. 4:**
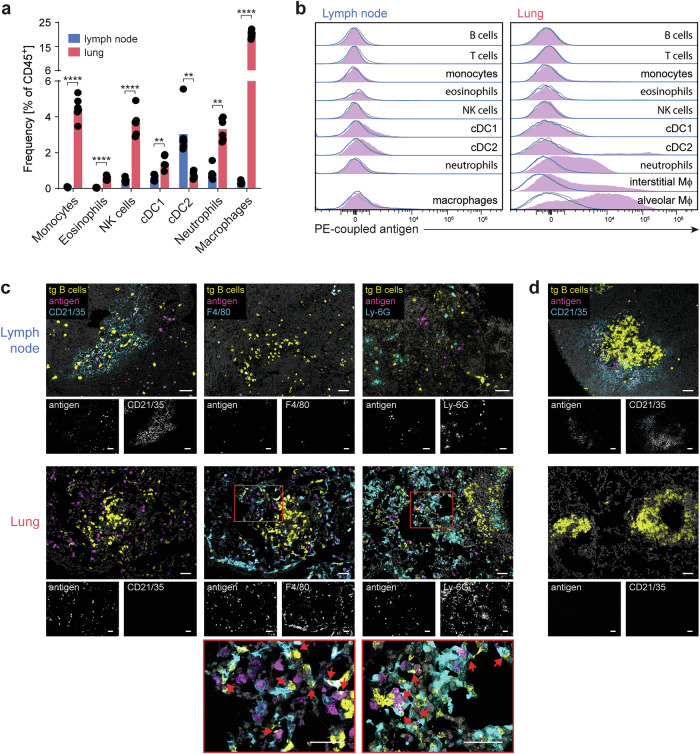
Compared with FDCs, macrophages and neutrophils are capable of presenting antigens to lung B cells but have much shorter kinetics in the lymph nodes. **A** Comparison of the frequencies of different APC populations in the lung and lymph nodes (see Supplementary Fig. S5 for gating). The symbols represent data from six mice from one representative experiment, and the bars represent the means. Three experiments with a total of 14 animals were performed. **B** Flow cytometry analysis of antigens retained on different APC populations in the lymph node and lung. The mice received fluorescent antigen four days (blue open histograms) or 24 h (purple filled histograms) prior to analysis. The gray lines show the autofluorescence background of the different cell types in the mice not challenged with the fluorescent antigen. Histograms are concatenated data from three animals. The data are representative of four experiments (12 mice), where the antigen was given 24 h prior to analysis. In only two of these four experiments, the additional group (6 mice), which received antigen 4 days prior to analysis, was included. **C** Immunohistological analysis of antigen-specific B cells and fluorescent antigen (see above) together with FDCs (CD21/35), macrophages (F4/80), or neutrophils (Ly-6G). The arrows at high magnification indicate B cells interacting with antigen-loaded macrophages or neutrophils. Data representative of two independent experiments with a total of 10 mice are shown. **D** Same analysis as in panel c; however, the last antigen application was four days before analysis. Representative tissue sections from two animals. The scale bars in all figures indicate 50 µm

Since FDC are absent in the lung, the question arises as to which other cell population could take over the function as antigen-presenting cells (APCs) for B cells in this organ. We identified a number of cell populations capable of capturing antigen in the form of immune or complement complexes (Fig. 4A and Supplementary Fig. S6), including monocytes, macrophages, dendritic cells, neutrophils, eosinophils, and NK cells [19]. Interestingly, on day 17, macrophages were the predominant APC population in the lungs, accounting for 20% of all CD45^+^ cells, and they were 63-fold more abundant in the lungs than in the lymph nodes. To assess which cell populations presented native antigens, we administered a fluorescent antigen (Fig. 4B and Supplementary Fig. S6), which in the lung was captured by almost all alveolar macrophages, a significant proportion of interstitial macrophages, and also by neutrophils and few type-2 conventional dendritic cells (cDC2s). In the lymph node, only a rather weak signal was obtained from dendritic cells and macrophages, which might reflect the lower antigen abundance in the lymph node.

Immunohistology revealed that FDC networks in the B-cell follicles stained positive for antigen in the lymph node, where B cells were in close contact with FDCs and that antigen-positive macrophages were located outside the B-cell follicles (Fig. 4C). In the lungs, FDCs are absent, and macrophages and neutrophils that take up the antigen are in contact with antigen-specific B cells. FDCs are well known for their ability to retain native antigens for periods of up to several months [20], whereas antigen presentation by phagocytes such as macrophages, which are more specialized in antigen processing, leads to limited antigen availability in time. Here, after antigen administration four days prior to analysis, we found that FDC still presented antigen in the lymph node, whereas antigen signals were completely lost in the lung (Fig. 4D).

**Fig. 5:**
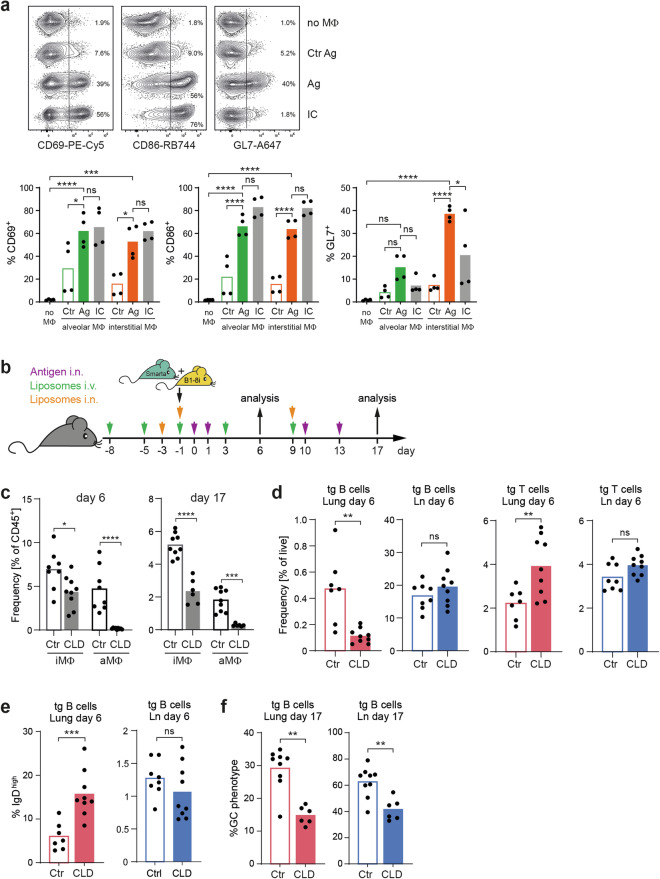
Antigen presentation by macrophages is critical for B-cell activation in the lung. **A** Interstitial and alveolar macrophages were sorted (for gating, refer to Supplementary Fig. S5) from the lung inflammation model on day 6. Two hours and 24 h before sacrifice, the mice received additional antigen, either Smarta peptide or NIP coupled to mouse serum albumin (Ag) or a conjugate without NIP (Ctr), intranasally. The macrophages were cocultured with naive B cells for 24 h before the B cells were analyzed for activation markers via flow cytometry. As a positive control, immune-complexed antigen (IC; antigen complexed with anti-NP IgM) was directly added to the cultures. As a negative control, B cells were cultured alone (no MΦ). The flow plots show representative examples of the expression of B-cell activation markers. The bar graphs represent the means of four culture wells (dots) from two independent experiments. **B** Experimental setup for the depletion of macrophages via i.v. and i.n. injections of clodronate or PBS control liposomes and antigen (interstitial macrophages are continuously replenished from blood monocytes and can therefore be removed by depletion of blood monocytes). The experiment was repeated two times with a total of eight control (Ctr) and nine clodronate (CLD) animals on day 6 and nine control and six clodronate animals on day 17. **C** Efficacy of the depletion of interstitial macrophages (iMΦ) and alveolar macrophages (aMΦ) on days 6 and 17. **D** Antigen-specific B and T-cell frequencies in the lung and lung-draining lymph nodes on day 6. **E** IgD expression on antigen-specific B cells from the lung and lymph nodes on day 6. **F** Fraction of antigen-specific B cells with a GC-like phenotype (GL7^+^ CD38^low^) on day 17. Pooled data from two independent experiments were used. In all the bar graphs, individual mice are symbolized by dots and bars indicating the means. ns, *p* ≥ 0.05; *, *p* < 0.05; **, *p* < 0.01; ***, *p* < 0.001; ****, *p* < 0.0001

To functionally demonstrate that macrophages capture native antigens on their surface and are effective APCs for B cells, we sorted alveolar and interstitial macrophages from the lung and cocultured them in vitro with naive antigen-specific B cells. Macrophages from mice that were immunized with cognate antigen were able to efficiently activate B cells in vitro, as shown by the upregulation of CD69 and CD86, downregulation of IgD, and expression of the B-cell activation and germinal center marker GL7. In contrast, macrophages from mice immunized with noncognate antigen were not able to efficiently activate B cells (Fig. 5A and Supplementary Fig. S7).

To confirm these results in vivo, we depleted macrophages with clodronate liposomes (Fig. 5B), which removed all alveolar and up to 50% of the interstitial macrophages (Fig. 5C). Although incomplete, this depletion had pronounced effects on early B-cell activation, in terms of an almost five-fold loss in antigen-specific B cells in the lung compared with the control (Fig. 5D). Furthermore, the downregulation of IgD was severely impaired (Fig. 5E). Notably, macrophage depletion did not affect B cells in the lung-draining lymph node, which is in line with the notion that FDC is the major antigen-presenting population in SLO. By analyzing macrophage-depleted mice on day 17, we observed a reduction in the number of GC-like B cells by a factor of two (Fig. 5F), further emphasizing the critical role of macrophages in B-cell activation in nonlymphoid tissue. In addition to presenting native antigens, macrophages are capable of processing antigens and presenting peptides in complex with MHC class II to T cells. Nevertheless, depletion of macrophages did not impair but even increased T-cell expansion in the lung (Fig. 5D), ruling out diminished T-cell help as a reason for impaired B-cell activation.

**Fig. 6:**
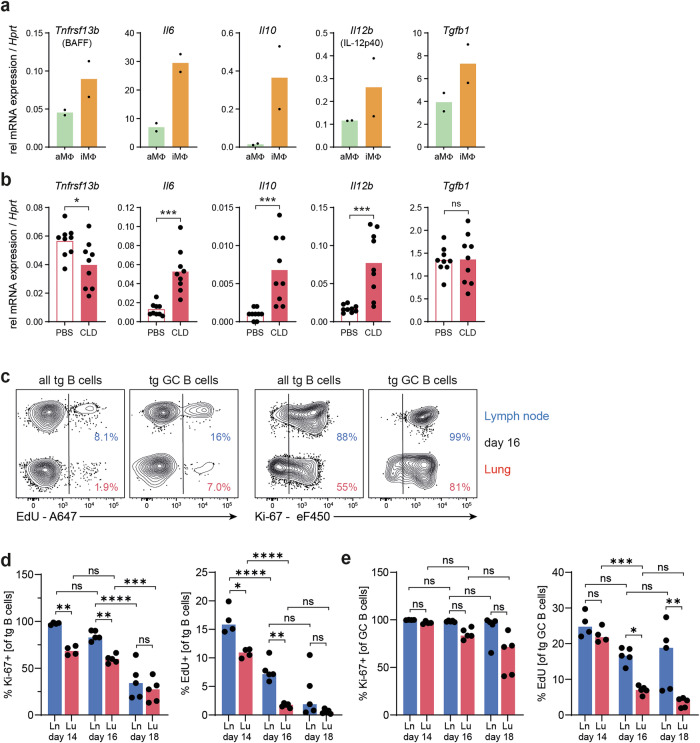
Macrophage depletion alters the lung cytokine milieu, and GC-like reactions in the lung contract faster. **A** Expression of the genes encoding BAFF, IL-6, IL-10, IL-12p40, and TGF-ß1 by alveolar and interstitial macrophages (for sorting, refer to Supplementary Fig. S5) was analyzed via qRT‒PCR on day 6. **B** After depletion of macrophages with clodronate, the expression of the abovementioned genes in whole lung tissue was analyzed. Analysis of lung and lung-draining lymph node B cell proliferation (Ki-67 expression and short-term EdU incorporation for two hours) at different time points by flow cytometry. This experiment was repeated two times, with a total of nine animals per time point. The results from one representative experiment with four animals on day 14 and five animals at days 16 and 18 are shown. **C** Representative flow cytometry plots from day 16. For gating of transgenic (tg) and tg GC B cells, see Fig. 1B. Percentages of Ki-67^+^ and EdU^+^ B cells within **D**, all antigen-specific B cells and **E**, transgenic B cells with a GC-like phenotype. In all the bar graphs, individual mice are symbolized by dots and bars indicating the means. ns, *p* ≥ 0.05; *, *p* < 0.05; **, *p* < 0.01; ***, *p* < 0.001; ****, *p* < 0.0001

In addition to their function as antigen-presenting cells, macrophages might also affect B cells through their production of survival and proliferation factors such as BAFF and IL-6 or other cytokines that direct their differentiation, such as IL-10, IL-12, and TGF-ß [1]. These factors are indeed produced by alveolar and interstitial macrophages from inflamed lungs (Fig. 6A). However, depletion of macrophages by clodronate did not substantially change or even increase the overall expression of these cytokines or survival factors in the lung (Fig. 6B), indicating that other cell populations can compensate for their production, most likely by neutrophils [21], which are increased in the lungs of clodronate-treated animals.

Next, to further assess B-cell proliferation, we applied short-term incorporation of 5-ethynyl-2′-deoxyuridine (EdU) and intracellular Ki-67 staining (Fig. 6C). Both methods revealed that the proliferative capacity of B cells was comparable in the lymph nodes and lungs directly after antigen challenge and decreased much faster in the lungs than in the lymph nodes (Fig. 6C). This difference was even more pronounced within the subpopulation of GC-like B cells (Fig. 6D). While lymph node-derived GC B cells maintained high EdU incorporation until day 18, proliferation rates rapidly decreased in the lung. These findings are in line with the lower expression of metabolic and cell cycle-associated genes in lung-derived GC-like B cells (Fig. 2D).

Taken together, in the lung, macrophages can replace FDCs in their function as presenters of native antigen to B cells. While the initial antigen levels in the lung are much higher than those in the lymph node are, the duration of antigen availability is highly restricted in the lung, and B cell proliferation rates are subject to rapid decay after antigen stimulation.

### B cell selection in the lung is efficient and occurs independently of processes in the lymph node

**Fig. 7:**
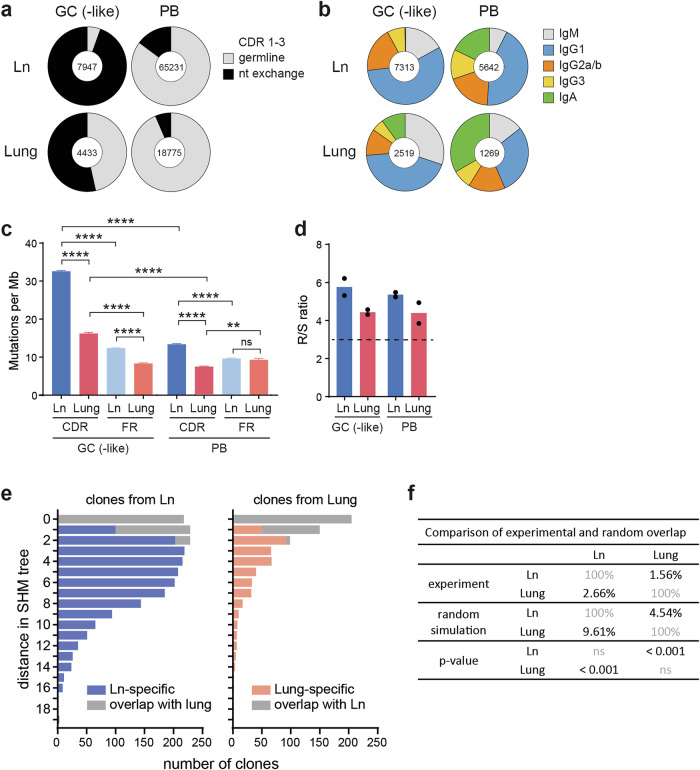
B cell selection in the lung is efficient and takes place independently of the draining lymph node. Antigen-specific B cells were isolated from the lung and the lung-draining lymph node (Ln) (two independent experiments with pooled organs from 10 mice each). B cells were further sorted into GC-like B cells and plasmablasts (PBs) via the markers shown in Fig. 1B, and heavy chain sequences were analyzed. Representative data from one experiment are shown. **A** Percentage of sequences containing at least one nucleotide mutation in the CDR. The numbers indicate unique sequences (UMIs) within each sample. **B** Distribution of different isotypes. The numbers indicate unique clones within each sample. **C** Average number of nucleotide mutations in the Ig heavy chain, separately analyzed for the CDR and framework (FR) regions. The error bars indicate the SEM. ns, *p* ≥ 0.05; **, *p* < 0.01; ****, *p* < 0.0001. **D** Ratio of amino acid substitutions (R) to silent (S) mutations (R/S) in the CDR. The dotted line indicates the VH186.2 sequence-specific calculated R/S value for random mutation without any selection. The dots represent two independent experiments. Antigen-specific B cells with a GC-like phenotype were isolated from the lungs and lymph nodes of a single mouse and analyzed via scRNA-seq. Clonal trees were generated from the heavy chain BCR sequence (compare Supplementary Fig. S9). **E** Number of clones within each tree level (0 = germline sequence). Clones shared between the lymph node and lung are displayed as gray bars, and clones unique to the lymph node or lung are displayed as blue and red bars, respectively. **F** Comparison of the experimentally observed clone overlap with the expected overlap if the clones were randomly mixed between the lung and lymph nodes. Only clones with more than one mutation were included in this analysis. The p values indicate whether the experimentally observed overlap is significantly lower than the expected overlap

The highly organized structure of the GC with dark and light zone separation is generally considered a prerequisite for efficient SHM of the BCR and selection of high-affinity antibody-producing clones [22]. To assess whether efficient B cell selection can occur in unstructured T cell/B cell infiltrates of the lung, we sorted antigen-specific GC-like B cells and plasmablasts (see Fig. 1B) and sequenced immunoglobulin heavy chains. As expected, more than 94% of the sequences obtained from lymph node-derived GC B cells carried at least one amino acid mutation in their complementarity-determining regions (CDRs) (Fig. 7A). Although fewer than in the lymph node, 54% of GC-like B cells in the lung presented mutations in their BCRs, indicating significant SHM in unstructured lung infiltrates. Within the plasmablast subset, only 15% and 7% of sequences were mutated in the lymph node and lung, respectively, suggesting that the majority of plasmablasts originated from extrafollicular reactions. Isotype class switching from IgM to IgG and IgA isoforms was as efficient in the lung as in the lymph node (Fig. 7B) and even more efficient in the lymph node and lung plasmablasts (92% and 85%, respectively). In both organs, IgG1 was the dominant isotype. Notably, IgA^+^ GC-like B cells were almost exclusively found in the lung but not in the lung-draining lymph nodes. This result was also confirmed at the protein level via flow cytometry (Suppl. Fig. S8). Compared with plasmablasts, GC-like B cells from the lung and lymph nodes presented a significantly greater mutation rate (Fig. 7C) and an accumulation of mutations in the CDR over the framework regions, whereas the mutation rate of GC-like B cells was significantly lower than that of B cells derived from the lymph nodes. A bias toward amino acid replacement (R) mutations over silent (S) mutations suggests that BCR hypermutation involves active selection for increased antigen binding rather than being completely random. The CDR R/S ratio for GC-like B cells from the lymph node and lung ranged between 4.3 and 6.2 in the two independent experiments (Fig. 7D), which is substantially greater than the VH186.2 sequence-specific calculated R/S value of 3.17 for random mutation without any selection [23].

Next, to further investigate the possibility of B-cell immigration from the lung-draining lymph node into the lung tissue after mutation onset, we analyzed cell shuttling between both organs by isolating GC-like B cells from the lungs and lymph nodes of a single mouse and analyzing SHM via single-cell BCR sequencing. The majority of sequences with only one mutation overlapped between the lung and lymph nodes due to random effects, whereas clones with two or more mutations were largely exclusive to the lung or lymph nodes (Fig. 7E and Supplementary Fig. S9). An in silico overlap simulation demonstrated that the observed overlap was significantly smaller than expected by randomization (Fig. 7F and Supplementary Fig. S10). The above findings from a single mouse were supported by two other experiments in which lung and lymph node B cells from a pool of 10 animals were analyzed by bulk BCR sequencing (Supplementary Fig. S9). Additionally, from these sequences, complete organ-specific clonal trees could be constructed, with almost no overlap for clones with more than one mutation.

Taken together, these data show that immunoglobulin class switching and SHM efficiently occur not only in the lymph node but also in the lung infiltrates and that BCR hypermutation occurs independently in the lung and lung-draining lymph nodes, without cellular trafficking between the two organs.

### B cells from the lung selectively lack high-affinity mutations and are clonally diverse

**Fig. 8:**
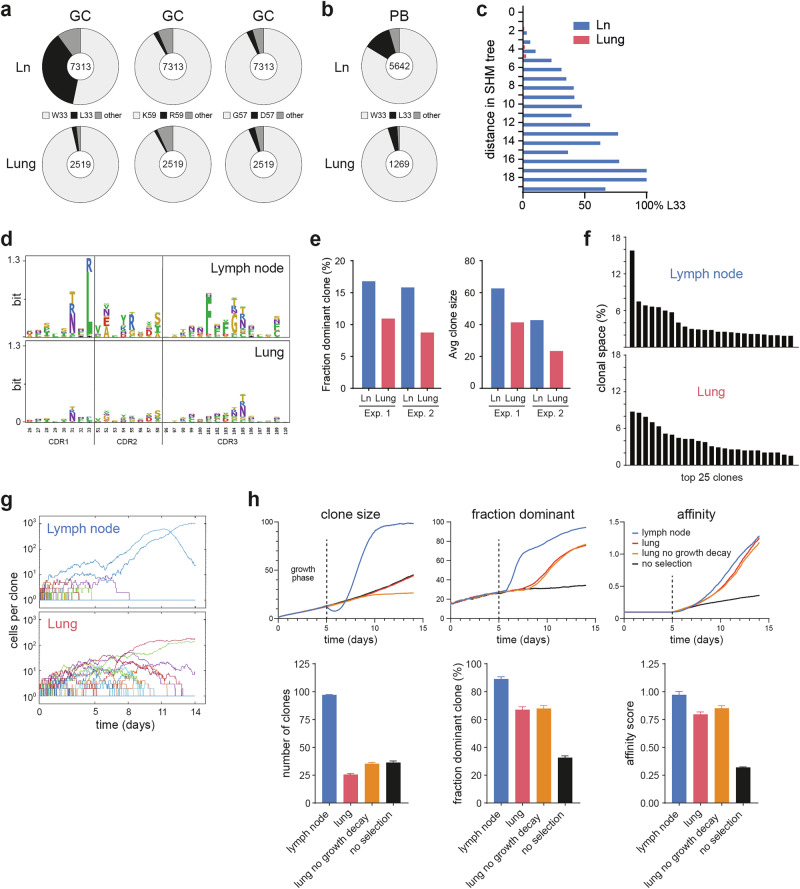
B cells from the lung selectively lack high-affinity mutations and have a broader clonal distribution. Analysis of amino acid mutations in the Ig heavy chain of GC-like B cells from the lung and lymph nodes. Representative data from one out of two bulk BCR sequencing experiments are shown, except for panel c, where sequencing data from the single-cell BCR experiment were used. **A** Percentage of GC-like B cell clones with the affinity-enhancing mutations W33L, K59R, and G57D. **B** Percentage of plasmablasts with the W33L mutation. **C** Percentage of clones with W33L mutation within each level of the SHM tree. **D** Substitution profiles of amino acid mutations in the CDR regions. The sequence logos display the frequency of amino acids other than the germline at each position. **E** Fraction of the dominant clone and average size of the GC-like B-cell clones among the top 25 clones. **F** Clonal space distribution of GC-like B cells from lung-draining lymph node and lung tissue. In silico model for B-cell expansion and affinity maturation. **G** Representative simulations of clonal expansion over time in the lymph node and lung settings. **H** Plots for the development of clone size, fraction of the dominant clone and affinity over time (top). Here, additional graphs for scenarios with no selection and no growth decay in the lung are shown. The bar graphs show the day 12 mean values (with SEM) over 100 independent simulations

For the immune response to the model antigen nitrophenol (NP) [24], several defined mutations within the heavy chain are known to confer increased binding affinity, the key mutation being an exchange of tryptophan for leucine at amino acid position 33 (W33L). This single mutation confers approximately ten-fold greater affinity than the germline mutation does and is therefore highly accumulated in B cells from NP immune responses [25]. Consistently, we found that more than one-third of all lymph node-derived GC B cells expressed leucine at position 33 of their heavy chain BCR (Fig. 8A), and another 10% of the clones exchanged tryptophan for other amino acids. In stark contrast, the W33L key mutation was almost absent in GC-like B cells from the lung. Similar results were obtained from the single-cell BCR analysis (Fig. 8C), and the clonal tree analysis also revealed that particularly lymph node clones with multiple mutations had leucine at position 33. The absence of this high-affinity mutation in the lung could not be explained by faster differentiation of high-affinity GC-like B cells to plasmablasts, since the W33L mutation was also underrepresented in this population (Fig. 8B). A general defect in the selection of high-affinity B cells was also ruled out, since two other well-described affinity-enhancing mutations (K59R and G57D [26]) were equally common in lymph node and lung GC-like B cells (Fig. 8A).

Next, we systematically screened all amino acid exchanges in the CDR (Fig. 8D). In addition to the W33L mutation, we identified other preferred mutations, such as Y101F in the CDR3, which was highly selected in lymph nodes but not in lung GC-like B cells, although it is unknown whether this mutation confers an affinity gain. The selective absence of high-affinity W33L clones, which normally dominate NP immune responses in SLO [25], is likely to alter the clonal composition of lung B cells compared with that of lymph node B cells. Indeed, the clonal distribution revealed that, in the lymph node, both the proportion of the dominant clone and the average clone size were greater than those in the lung (Fig. 8E), which was also evident in the clonal space distribution of the top 25 clones (Fig. 8F). In the lymph node, one strongly dominant clone (with a W33L mutation) occupied 16% of the clonal space, whereas in the lung, the size of the top 25 clones was more evenly distributed.

### An in silico model reveals efficient yet altered B-cell selection in the absence of GC-specific processes

In germinal centers, B-cell selection is driven by a complex mechanism that includes strictly limited T-cell help and the recirculation of B cells between the light and dark zones, in addition to selection by affinity to antigens. Previous experimental and computational studies have linked these processes to optimizing the efficiency of SHM [5, 27, 28]. To assess the ability of nonorganized lung infiltrates to generate an antigen-specific B-cell pool, we established a stochastic mathematical model of B cell affinity maturation considering two different scenarios (Supplementary Fig. S11): in the lymph node, cells shuttle between the light and dark zones and receive signals via antigen recognition and T cells only in the light zone; in the lung, B cells have constant access to T cell help and antigens, which, however, disappears faster (parameterized by data from Fig. 6C–E). In both cases (Fig. 8G), typical simulations revealed that the onset of competition on day five caused a separation into a small number of quickly proliferating, high-affinity clones, while the majority of the clones were prone to extinction. Here, in the lymph node scenario, two dominant clones emerged after seven days, whereas in the lung scenario, more clones expanded equally, and there was less variation in clone size after two weeks. Statistical analysis of our simulations across 100 runs (Fig. 8 H) confirmed a lower degree of clonal segregation in terms of a fourfold reduction in the average clone size and a lower proportion of the dominant clone in the lung than in the lymph node scenario, whereas only minor differences in affinity maturation were present. Notably, the described pattern of a pronounced but lower degree of clonal segregation in the lung than in the lymph nodes is in good agreement with our experimental observations (Fig. 8E).

Overall, we found that efficient B-cell affinity maturation is not limited to germinal center organization, although properties such as clonal segregation and dominance of clones with very high affinity are amplified in germinal centers. Furthermore, model simulations indicated that structural elements of GC dynamics, such as light-zone/dark-zone shuttling and pronounced Tfh cell support, can explain the strong and rapidly established clonal segregation in germinal centers.

## Discussion

The complex microarchitecture of SLOs, or at least the presence of ELS, such as iBALT, is widely accepted as a prerequisite for the generation of high-affinity plasma cells or memory B cells, which can migrate to inflamed nonlymphoid tissue after differentiation toward effector or memory cells. Even influenza-specific memory B cells in the lung are typically not generated locally but are recruited from the circulating memory pool to iBALT structures [29, 30]. Nevertheless, it has long been known that naive T and B cells recirculate through nonlymphoid tissues such as the lung, liver, kidney, pancreas and intestinal lamina propria [17, 18], but the biological significance of the migratory behavior of naive cells has been enigmatic. In our study, we showed that naive B cells can be rapidly activated directly in lung tissue with the help of antigen-bearing macrophages. Even at later time points, we did not find any evidence for trafficking of B cells between lung-draining lymph nodes and lung tissue. Importantly, we found that distinct and complete clonal trees of SHM can be built with B cells from each organ.

An important open question in this context relates to how presentation of native antigen to B cells occurs in the lung, as the lung infiltrates in our mouse model are devoid of FDCs. Here, we found that macrophages and neutrophils are able to retain native antigens but, unlike FDCs, do not provide long-term deposits of antigens. Neutrophils generally have a rather short life span of only 12.5 hours in mice [31]. The presentation of native antigens by macrophages is most likely limited by their preferential degradation of the antigen. Nevertheless, they play a key role in B-cell activation in the lung, as depletion of interstitial and alveolar macrophages strongly impaired B cell activation, and coculture of antigen-bearing lung macrophages with antigen-specific B cells resulted in effective B-cell activation. Owing to their localization, interstitial macrophages are more likely candidates for the activation of B cells. However, it has recently been shown that lung-resident memory B cells migrate into alveolar spaces for reactivation, where they interact with alveolar macrophages [32]. To date, macrophages have been mainly discussed as presenters of peptide/MHC class II complexes for the activation of T cells in inflamed tissues. However, in our study, the expansion of antigen-specific T cells in the lung was even promoted in the absence of macrophages. This could be due to the inhibitory function of alveolar macrophages, which have been reported to suppress T-cell proliferation in vitro [33]. Alternatively, cDCs in the lung may have an advantage in antigen presentation when the much larger population of lung macrophages no longer competes for antigen uptake.

As a central finding from our study, B-cell activation and differentiation in unstructured nonectopic infiltrates resulted in efficient SHM. Previously, GC-independent affinity maturation of B cells was described only for SLO by Shlomchik and colleagues in extrafollicular foci in the spleen of autoimmune mice [34]. SHM and selection have also been shown to occur in immune responses to certain bacteria that actively suppress the formation of GC structures, such as *Salmonella typhimurium* or *Ehrlichia muris* [35, 36]. Strikingly, the W33L key mutation, which is a single mutation that confers an approximately ten-fold greater affinity to the NP-specific BCR [25], is almost completely absent in the lung. A likely explanation is that lung B cells undergo fewer rounds of proliferation due to the faster decay of antigens and IL-21 production by Tph cells in the tissue than in the lymph nodes. In the clonal tree for lymph node B cells, the W33L mutation is preferentially found in cells with six or more mutations, which are underrepresented in the lung. Interestingly, memory B cells from autoimmune patients, which are likely selected by Tph cells in inflamed tissue rather than by Tfh cells in SLO, have been reported to have a lower degree of SHM than healthy donors. This has been shown for double-negative B cells from lupus patients [37], isotype-switched B cells from rheumatoid arthritis patients [38], and memory B cells from ulcerative colitis patients [39].

Another important difference in B cells selected in the lung and not a classical GC reaction is the broader distribution of individual clones. This less stringent selection, permanent availability of T cell help and lack of dark and light zone segregation increase the risk of expansion of autoreactive B cells. Indeed, there are many indications that the lung may be an inductive site for systemic autoimmune diseases. The constant low level of inflammation owing to constant exposure to exogeneous antigens favors the recruitment of T and B cells into the lung tissue, and the less controlled environment for SHM could easily turn a B cell directed against a harmless environmental antigen into an autoreactive B cell. In rheumatoid arthritis patients, B cells autoreactive against citrullinated proteins are found in the lung several years before any joint involvement [4]. In a mouse model, T cells primed in the intestinal mucosal system have been shown to subsequently drive autoimmune arthritis [40]. In addition to the ability of the lung as an induction site for autoreactive B cells migrating to distinct sites, lung infiltrating B cells can also be a major factor for local tissue destruction. We recently showed that in the lungs of sarcoidosis patients, B cells form large infiltrates that give rise to plasmablasts, which locally produce large amounts of immunoglobulins [12].

On the other hand, a broader and less affine BCR repertoire may be advantageous to combat viral escape mutants. This has been shown in mouse models of West Nile virus infection, where low-affinity memory B cells were superior to high-affinity plasma cells in producing neutralizing antibodies against mutant viral epitopes [41], and in influenza, where interruption of the GC response by rapamycin promoted the generation of antibodies with lower affinity but cross-protective properties [42]. In addition, low-affinity memory B cells are preferentially recruited into new GC reactions upon re-encounter with the antigen, where they can further optimize their BCR [41].

Hence, strategies to promote Tph-driven B cell differentiation directly in the lung tissue are highly desirable for vaccination purposes, especially since isotype switching toward IgA occurs almost exclusively in lung GC-like B cells. IgA is the only immunoglobulin subclass that can be secreted directly into the airways [2]. There, it can neutralize pathogens prior to tissue entry and thereby confer sterile immunity.

## Materials and methods

### Mice

All the animal experiments were approved by the local authorities and performed in accordance with the German animal protection laws. The mice were bred under specific pathogen-free conditions in the DRFZ animal facility at the Federal Institute for Risk Assessment, Berlin, and the central animal facility of the University Hospital Schleswig-Holstein in Kiel or bought from Charles River Laboratories (Sulzfeld, Germany). C57BL/6NCrl and CD28 knockout mice (Jax stock 002666) were used as recipients for adoptive transfer experiments. The advantage of CD28 knockout recipients is that they cannot mount an endogenous T cell response, i.e., the T-cell help comes only from the transferred Smarta cells. However, this does not result in any differences for this model. Smarta T-cell receptor transgenic mice [43] were additionally backcrossed to congenic B6PL mice (Jax stock 000406; Thy-1.1^+^) to track cells after adoptive transfer. To analyze IL-21 production directly ex vivo, the strains were also crossed with an IL-21-FP635 reporter strain [44]. For the transfer of antigen-specific B cells, nitrophenol (NP)-specific BCR knock-in B1-8i mice [45] were crossed with κ-L chain knockout mice [46] to ensure the NP specificity of all B cells, and with Ly-5.1 mice (Jax stock 002014; CD45.1^+^) to track cells in vivo. Both sexes of the mice were used at an age between 8 and 20 weeks.

### Basic adoptive transfer lung inflammation model

A total of 1.25 × 10^5^ Smarta T cells were cotransferred with 5 × 10^5^ follicular B1-8i B cells into recipient mice via intravenous injection. To induce lung inflammation, the mice were repeatedly challenged intranasally with 10 µg of cognate antigen (Smarta peptide and iodinated nitrophenol (NIP) coupled to mouse serum albumin as a nonimmunogenic carrier [16]) and 5 µg of LPS (Sigma‒Aldrich, St. Louis, MO, USA) as an adjuvant on days 0, 1, 10, and 13.

### Analysis of antigen-presenting cells

To visualize the antigen via flow cytometry and histology, in some experiments, the mice received a NIP and Smarta peptide conjugate with phycoerythrin as the carrier on day 13 or 24 hours before sacrifice (10 µg for flow cytometry and 100 µg for immunohistology).

### Analysis of early B-cell activation in the lung

To increase the number of endogenous B cells in the lung, the recipient mice received 5 µg of LPS intranasally three days prior to immunization (day -3). Adoptive transfer of transgenic T and B cells was performed on day -1; B1-8i B cells were sorted for naive CD62L^high^ cells via magnetic sorting (Miltenyi Biotec, Gladbach, Germany), and the number of transferred cells was increased to 2 x 10^6^ B cells and 5 × 10^5^ Smarta T cells. One day later (day 0), the mice were intranasally immunized with the cognate antigen as described above. One group was additionally injected i.p. with 20 µg of FTY720 (Sigma‒Aldrich). This injection was repeated 12 h later. Analysis was performed 24 h after immunization. For the 72-hour analysis, the mice were not pretreated with LPS, and the FTY injection was repeated after 24 and 48 h.

### Depletion of macrophages

To deplete alveolar macrophages, C57BL/6NCrl mice received 200 µg of clodronate liposomes (Liposoma BV, Amsterdam, Netherlands) intranasally on days -3, -1, and 9 according to the adoptive transfer lung inflammation model described above. To deplete interstitial macrophages, the mice were additionally treated with 750 µg of clodronate liposomes intravenously on days -8, -5, -1, 3, and 9. PBS liposomes were used as controls.

### Cell isolation

Mice were either perfused with PBS to remove blood leukocytes from the lung or received 3 µg of fluorophore-coupled antibodies against CD3 and B220 (and CD11b for some experiments) shortly before sacrifice to discriminate vascular and extravascular T and B cells [47] (or APCs for some experiments). For analysis of airway cells, bronchoalveolar lavage was performed by flushing the lungs twice via the trachea with 1 ml of PBS. The lungs were removed, and bronchial lymph nodes were dissected from the lung tissue under a stereomicroscope. Single-cell suspensions from lung tissue were prepared via a gentleMACS Dissociator (Miltenyi Biotec) and digested with 0.5 mg/ml collagenase D and 33 µg/ml DNase I (both from Roche) for 25 min at 37 °C [13]. The lymph nodes were passed through a 70 µm sieve, except for the experiments comprising APC analysis, where they were digested similarly to the lungs. The cells were counted with ViaCount solution via either a Guava Muse capillary flow cytometer or an Aurora flow cytometer (all from Cytek Biosciences, Fremont, CA, USA).

### Flow cytometry

Single-cell suspensions of the lymph nodes or lungs were stained with different combinations of fluorophore-conjugated antibodies (Supplementary Table S1). All the antibodies were tested and titrated before use. After blocking Fcγ receptors with 20 µg/ml anti-CD16/32, the cells were stained for 30–40 min on ice. Staining with biotinylated antibodies required an additional step with fluorophore-labeled streptavidin for 5 min. Dead cells were excluded by either adding DAPI immediately before analysis or using a fixable live/dead stain (Pacific Orange, Alexa Fluor 350, or Alexa Fluor 700 succinimidyl ester; Thermo Fisher Scientific, Waltham, MA, USA) prior to the staining procedure [48]. For the intracellular staining of Bcl-6 or Ki-67, the Transcription Factor Staining Buffer Set from eBioscience (San Diego, CA, USA) was used. For detection of EdU incorporation, the EdU Cell Proliferation Kit from Thermo Fisher was used. The mice received 1.5 mg of EdU by i.p. injection two hours before sacrifice. Flow cytometry analysis was performed on an LSR Fortessa (Becton Dickinson, Franklin Lakes, NJ, USA) or Cytek Aurora (Cytek Biosciences) flow cytometer, and the data were analyzed with FlowJo v10 software (Tree Star Inc., Ashland, OR, USA). In all figures, antigen-specific T cells were identified as live CD19^-^ CD4^+^ Thy-1.1^+^ and antigen-specific B cells as live CD4^-^ CD19^+^ CD45.2^-^ CD45.1^+ cells^.

### Immunohistology

The lungs were filled with 50% TissueTek OCT compound (Sakura Finetek, Tokyo, Japan) in PBS via the trachea and snap frozen in ice-cold isopentane. Cryostat sections (8 µm) were cut and fixed with acetone. After peroxidase inactivation and blocking with casein solution (Vector Laboratories, Burlingame, CA, USA), the sections were stained with the antibodies listed in Supplementary Table S2. Tyramide signal amplification was performed via peroxidase-coupled anti-FITC or anti-digoxigenin antibodies and Alexa Fluor 488-, 555-, or 647-coupled tyramide (Thermo Fisher Scientific). Nuclei were counterstained with DAPI, and slides were mounted with Fluoromount (Sigma‒Aldrich). Images were captured on a Carl Zeiss (Oberkochen, Germany) LSM 880 laser scanning microscope with ZEN 2.3 software.

### Macrophage/B-cell coculture

The macrophages were isolated from the lung inflammation mouse model on day 6. The mice received additional cognate (Smarta peptide + NIP coupled to mouse serum albumin) or noncognate (Smarta peptide coupled to only mouse serum albumin) antigen 24 h and two hours before sacrifice. The alveolar and interstitial macrophages were sorted on an ARIA Fusion cell sorter according to the gating scheme shown in Supplementary Fig. S5. Naive antigen-specific B cells were isolated from the spleens of B1-8i mice via CD43 depletion, followed by selection of CD62L^high^ cells via magnetic cell sorting. The macrophages and B cells were cocultured at a 1:3 ratio in 96-well round-bottom plates in complete RPMI 1640 medium at 37 °C and 5.2% CO_2_. As a positive control, 10 µg/ml cognate antigen complexed with anti-NP-IgM (clone 267.7 µ, kind gift from Klaus Rajewsky, Berlin) was added. Cultures were analyzed by flow cytometry after 24 and 48 h.

### Single-cell RNA sequencing

Antigen-specific B cells from the adoptive transfer system were isolated from the lung and lung-draining lymph nodes on day 17 and enriched for CD45.1-expressing cells via magnetic sorting. For higher purity, cells were sorted on an ARIA II or ARIA Fusion cell sorter (Becton Dickinson, Franklin Lakes, NJ, USA) as CD4^-^ CD8^-^ CD19^+^ CD45.1^+^ CD45.2^-^ total B cells for the transcriptome and further as CD138^-^ CD38^low^ GL7^+^ for the BCR experiment. The cells were separated on a 10x Genomics (Pleasanton, CA, USA) Chromium Controller, and libraries were prepared via the Chromium Next GEM Single Cell 3’ v2 Kit or the Chromium Next GEM Single Cell 5’ v2 Kit in combination with the Single-cell Mouse BCR Amplification Kit. Sequencing was performed on an Illumina (San Diego, CA, USA) NextSeq 500 or NextSeq 2000 system using a high-output v2 flow cell.

### Single-cell RNA data analysis

The raw sequence reads from the experiment depicted in Fig. 2 were processed via Cell Ranger (version 5.0.0; 10x Genomics). Demultiplexing, mapping, detection of intact cells and quantification of gene expression were performed via Cell Ranger’s count pipeline with default parameter settings, with an expected number of 3000 cells per sample and a reference-cellranger-mm10-1.2.0 as a reference. This led to 2565 cells (1440 median genes per cell, 56,303 mean reads per cell) for the lung sample and 3788 cells (1311 median genes per cell and 48,723 mean reads per cell) for the lymph node sample. Next, Cell Ranger aggr was used to merge the libraries without size normalization. The samples were further analyzed in R (version 4.1.2) via the Seurat package (version 4.0.5) [49]. In particular, samples were read via Read10x and CreateSeuratObject and normalized by NormalizeData with LogNormalization as normalization.method and a scale factor of 10,000. A uniform manifold approximation and projection (UMAP) was computed via FindVariableFeatures with 2000 variable genes and vst as selection.method, ScaleData, RunPCA to compute 50 principle components and RunUMAP with dimensions of 1:30. Transcriptionally similar clusters were identified via shared nearest neighbor (SNN) modularity optimization via FindNeighbors with pca as the reduction and 1:30 dimensions and FindCluster with resolutions ranging from 0.1--1.0 in 0.1 increments. The optimal resolution was chosen by visual inspection of the percentage of mitochondrial genes, UMI counts, number of identified genes and expression of typical marker genes for GC B cells and plasmablasts projected on the UMAP. Clustering with a resolution of 0.1 was judged to best separate the B-cell subsets and define low-quality cells. Cells in cluster 0 (GC-like B cells) were extracted and reanalyzed separately as described above. By visual inspection, resolutions of 0.4 and 0.2 were considered to best reflect their transcriptional community structure.

Similarly, the raw sequence reads from the experiment depicted in Fig. 7e were processed via Cell Ranger (version 7.1.0; 10x Genomics) with the default parameter settings, and refdata-gex-mm10-2020-A was used as a reference. This led to 1471 cells (2818 median genes per cell, 159,367 mean reads per cell) for the lung and 4048 cells (2741 median genes per cell and 64,047 mean reads per cell) for the lymph node sample. Next, Cell Ranger aggr was used to merge the libraries without size normalization. UMI counts for immunoglobulin genes and pseudogenes as defined by the gene_biotype were removed for further analysis in R. A UMAP was computed as described above.

### Analysis of differentially expressed genes and pathway analysis

FindAllMarkers with the default parameter settings were used to define DEGs between clusters. Bubble plots are based on z-transformed median expression values of the top differentially expressed genes as determined by the fold change and a Bonferroni corrected *p* value (Wilcoxon rank sum test) below 0.01. Gene set enrichment analysis (GSEA) was performed as previously described [50]. In particular, a pseudo bulk GSEA was performed for each cell on the basis of the difference from the mean of log normalized expression values of all cells in the analyzed set as a preranked list and 1000 randomizations. Significant up- or downregulation was defined by an FDR ≤ 0.25 and a normalized *p* value < 0.05, as recommended [51]. For visualization, the NESs for significant cells were plotted. GSEA was performed for cluster 0, GC-like B cells and BCR cells via hallmark gene sets, REACTOME, and KEGG, which were obtained from the MSigDB collection [52]. Pathway enrichment is defined by the ratio of significant cells in the lung and lymph nodes.

### Single-cell BCR analysis

The raw sequence reads were processed via CellRanger (version 7.1.0). Vdj was used with the default parameter settings for demultiplexing and assembly of the B-cell receptor sequences via refdata-cellranger-vdj_GRCm38_alts_ensembl-mouse-2.2.0 as a reference. In the case of multiple contigs, the most abundant, productive and fully sequenced contig for the heavy and light BCR chain was used. This led to the detection of 681 cells with 639 IGHV1-72 heavy chain annotations in the lungs and 2305 cells with 2124 IGHV1-72 heavy chain annotations in the lymph nodes. Overlap statistics were performed as described for bulk BCR sequencing.

### Bulk BCR sequencing

Antigen-specific B cells from a day 18 immune response were isolated from the lymph nodes and lungs and magnetically enriched as described above. Plasmablasts were sorted as DAPI^-^ CD4^-^ CD8^-^ CD45.1^+^ CD45.2^-^ CD19^low^ CD138^+^ and GC B cells as DAPI^-^ CD4^-^ CD8^-^ CD45.1^+^ CD45.2^-^ CD19^+^ CD38^low^ GL7^+^ on an ARIA II cell sorter directly into TRIzol LS reagent (Invitrogen, Waltham, MA, USA). RNA was isolated via the Qiagen (Hilden, Germany) RNeasy Micro Kit. Heavy chain immunoglobulin BCR libraries were prepared according to the protocol of Chudakov [53] and sequenced on an Illumina MiSeq system (600 cycle v3 kit).

### Bulk BCR data analysis

The raw sequence reads were demultiplexed with MIGEC-1.2.4a Checkout -cute and assembled with AssembleBatch --force-collision-filter and --force-overseq of 3 [54]. The resulting BCR heavy chains were classified according to the presence of isotype-specific sequences: CAAATGTCTTCCCCC for IgM, CCATCTACCCACTGA for IgA and N1ACAN2CCCCATCN3GTCTATCC for IgG, allowing for one mismatch. The IgG sequences were further subdivided into IgG1 when G, C and T were present at positions N1, N2 and N3; A, G and G into IgG2a; A, C and A for IgG2b; and A, G and T for IgG3. For the annotation of the framework (FR) and complementarity determining regions (CDR) and the determination of mutations, sequences were aligned pairwise to the Vh186.2-IgG1 sequence via the Needleman‒Wunsch algorithm. The FR and CDR of the Vh186.2-IgG1 sequence were determined via the IMGT Immunoglobulin Web Portal [55]. Silent mutations and replacement mutations were defined on the basis of the alteration of the corresponding amino acid. Mutations were normalized to the corresponding sequence length.

Lineage trees were computed with GLaMST [56] using the FR1 to FR4 sequences and the Vh186.2 sequence as the root input. Statistics on the overlap of repertoires between different samples were based on the presence of identical clonal sequences in different samples. A clonal sequence was defined by the FR1-FR4 sequence. To assess the likelihood of the observed overlap, the overlap was compared to 1000 randomly generated overlaps by shuffling all BCR sequences among the samples. Repertoire overlap statistics show the proportion of the FR1--FR4 sequences in each sample that were identified in the rowwise sample. Sequence logos of the CDR were computed as described by Schneider et al. [57] while considering the UMI counts.

### Analysis of cytokines and survival factors by qRT‒PCR

Total lung RNA was isolated via the Zymo Research (Orange, CA, USA) Quick-RNA Miniprep Kit. Organs were directly homogenized in lysis buffer supplemented with 0.5% Antifoam Y-30 (Sigma‒Aldrich) via a gentleMACS dissociator and M-tubes (Miltenyi Biotec). Alveolar and interstitial macrophages were sorted directly into TRIzol LS (Thermo Fisher Scientific) on an ARIA Fusion cell sorter according to the gating scheme shown in Supplementary Fig. S5. RNA was isolated via the Qiagen miRNeasy Micro Kit. cDNA was synthesized via a Roche (Penzberg, Germany) high-capacity cDNA synthesis kit. qPCR analysis was performed in duplicate on an Applied Biosystems (Carlsbad, CA, USA) QuantStudio 5 system via TaqMan Gene Expression Assays (Applied Biosystems, Supplementary Table S3).

### In silico model for BCR affinity maturation

We described BCR affinity maturation as a stochastic birth–death mutation process by adapting and extending a mathematical model [58]. In brief, both cell proliferation and cell death are controlled by a cell-intrinsic fitness score, which is subject to stochastic modification in every new generation, and we developed specific lymph node and lung scenarios on the basis of experimental data. The model is implemented in a straightforward stochastic simulation scheme in MATLAB, and the parameters were adopted from [58] and modified to reflect the shorter selection time period observed in our experiments (Supplementary Table S4).

In the model, each clone is initialized with a single cell of moderate fitness, described by a scalar weight or fitness score ${w}_{0}\in ({{\mathrm{0,1}}})$. Clones can expand via cell division or become extinct when their last member undergoes apoptosis. Both cell division and cell death follow a Poisson process. At each cell division, the fitness evolves via random mutation according to a diffusion process, ${w}_{i}={w}_{{parent}}+{N}(0,\sqrt{2D})$. The birth and death rates *λ*_*i*_ and *δ*_*i*_ of each cell $i\in [1,\ldots ,M]$ depend on ${w_{i}}$ as follows:

1 $${\lambda }_{i}=\frac{{\lambda }_{0}{{f}}_{\lambda }\left({w}_{i}\right)}{\bar{f}_{\lambda}}$$

2 $${\delta }_{i}=\frac{{\delta }_{0}{f}_{\delta }\left({w}_{i}\right)}{{\bar{f}_{\delta }}}+\left({\lambda }_{0}-{\delta }_{0}\right) \frac{M}{C}$$

Here,$\quad {\bar{f}_{\lambda }}={\sum }_{i}{f}_{\lambda }({w}_{i})/M$ and ${\bar{f}_{\delta }}={\sum }_{i}{f}_{\delta }({w}_{i})/M$ are the population-averaged growth and death rate modulations, respectively. The above definitions ensure that population averages are conserved, that is, $\left\langle {\lambda }_{i}\right\rangle ={\lambda }_{0},\,\left\langle {\delta }_{i}\right\rangle ={\delta }_{0}+\left({\lambda }_{0}-{\delta }_{0}\right)M/C$, independent of the form of the weighting functions *f*_*λ*_ and *f*_*δ*_. The death rate is further modulated by a term proportional to the ratio of the total cell number *M* and an assumed maximal capacity *C* of the GC to ensure that cells cannot grow without bounds even at very high affinity.

As an extension and generalization of the model by Amitai et al. [58], we define the functions ${f}_{\lambda }$ and ${f}_{\delta }$, which represent birth and death selection, respectively, in the following form of Hill-type equations:

3 $${f}_{\lambda }\left({w}_{i}\right)=1+\left({g}_{\lambda }-1\right)\frac{{{w}_{i}}^{h}}{1+{{w}_{i}}^{h}}$$

4 $${f}_{\delta }\left({w}_{i}\right)=\frac{1+{{w}_{i}}^{h}}{1+{g}_{\delta }\quad {{w}_{i}}^{h}}$$

This definition ensures that ${f}_{\lambda }\left(0\right)={f}_{\delta }\left(0\right)=1$ so that cells with small fitness values evolve with unchanged growth and death rates ${\lambda }_{0},\,{\delta }_{0}$. At ${w}_{i}\gg 1$, the growth rate tends toward ${g}_{\lambda }$, and the death rate tends toward $1/{g}_{\delta }$. Thus, the parameters ${g}_{\lambda }$ and ${g}_{\delta }$ are the maximal fold changes due to the fitness advantages of the growth and death rates, respectively.

To analyze our experimental results and specifically compare clonal selection in lymph nodes versus secondary lymphoid organs, we considered three different simulation scenarios as follows:

#### Growth phase

In line with [58], we assume that for an initial time period *T*_*g*_, the number of responsive cells is too small for competition among them; therefore, all the cells are able to grow independently of their individual weights. That is, instead of Eqs. 3, 4 above, we set ${f}_{\lambda }\left({w}_{i}\right)={f}_{\delta }\left({w}_{i}\right)=1$ for all ${w}_{i}$.

#### Lung

After the growth phase, the cells enter the competition phase for a time period *T*_*c*_. Both birth and death selection occur for all cells, according to Eqs. 1–4.

#### Growth decay in the lung

In addition to the processes of scenario (ii), a decay of the average growth rate ${\lambda }_{0}$ is considered after time ${T}_{{dec}}.$ Specifically, we use a new value ${\widetilde{\lambda }}_{0}$ in Eq. 3 above, and we set $${\widetilde{\lambda }}_{0}=\left\{\begin{array}{c}\left({\lambda }_{0}-{\delta }_{0}\right){e}^{\kappa \left(t-{T}_{{dec}}\right)}+{\delta }_{0}\,,\,t > {T}_{{dec}}\\ {\lambda }_{0}\,,{otherwise}\end{array}\right.$$. This form of the decay function ensures that the average growth rate does not become smaller than the average death rate ${\delta }_{0}$. The decay rate $\kappa$ is determined on the basis of the experiment shown in Fig. 6c–e.

#### Lymph node

In contrast to the lung, the lymph node is divided into the dark zone (DZ) and the light zone (LZ). In our model, cells stochastically transit from the DZ to the LZ and from the LZ to the DZ according to a Poisson process with rates ${k}_{{DZLZ}}$ and ${k}_{{LZDZ}}$, respectively (Supplementary Table S4). Only DZ cells can divide. Therefore, to attain comparable average growth rates, we modified the growth rate by a factor of ${\alpha }_{{LN}}=2$, which accounts for the observed fraction of time that each cell spends in the DZ on average (50%, which is in agreement with the experimental data). Specifically, we use a new value ${\widetilde{\lambda }}_{0}$ in the formalism below, and we set ${\widetilde{\lambda }}_{0}={\alpha }_{{LN}}{\lambda }_{0}$. Apart from proliferation in the DZ, we account for processes in the LZ that include receiving T cell help, both boosting their growth capacity and reducing their death rate. Together, these processes are described as follows:

Cells in the DZ: *λ*_*i*_ = *const; δ*_*i*_* = δ*_0_.

DZ-to-LZ transition: Update *δ*_*i*_ according to Eqs. 2 and 4.

Cells in LZ: *λ*_*i*_ = 0; *δ*_*i*_ = *const*.

LZ-to-DZ transition: Update *λ*_*i*_ according to Eqs. 1 and 3.

Notably, the growth rate is only updated upon re-entry into the DZ, not at each cell division, as in the lung scenario, because T-cell help is indispensable for LZ cells to exploit their fitness advantage.

### Statistical analysis

The data were analyzed via GraphPad Prism 10. No data exclusion criteria were used. After checking for a normal distribution (Shapiro‒Wilk test), significant differences were determined via ordinary one-way ANOVA, the Kruskal‒Wallis test, unpaired t test, or the Mann‒Whitney U test.

## Supplementary information

Supplemental Material

## Data Availability

Single-cell RNA-seq data and bulk BCR sequencing data are deposited with the National Center for Biotechnology Information (GEO), accession numbers GSE274347 and GSE274693. No unique reagents were generated in this study.
