# Supplementary material for: Activation and maturation of antigen-specific B cells in nonectopic lung infiltrates are independent of germinal center reactions in the draining lymph node
Source: Cell Mol Immunol. 2025 Apr 11;22(6):612–27. doi: 10.1038/s41423-025-01285-8 (PMC12125278; doi:10.1038/s41423-025-01285-8)
Supplement: Supplementary file 1 — Supplemental Material [file 41423_2025_1285_MOESM1_ESM.pdf]

Supplementary Material for

Activation and maturation of antigen-specific B cells in non-ectopic lung infiltrates are independent of germinal center reactions in the draining lymph node

Sarah-Sophie Schacht, Josefine Graffunder, Pawel Durek, Jonas Wehrenberg, Annette Siracusa, Charlotte Biese, Mir-Farzin Mashreghi, Kevin Thurley, Laura Bauer, Andreas Hutloff

Supplementary Material

Supplementary Figures S1 - S11

Supplementary Tables S1 - S4

References

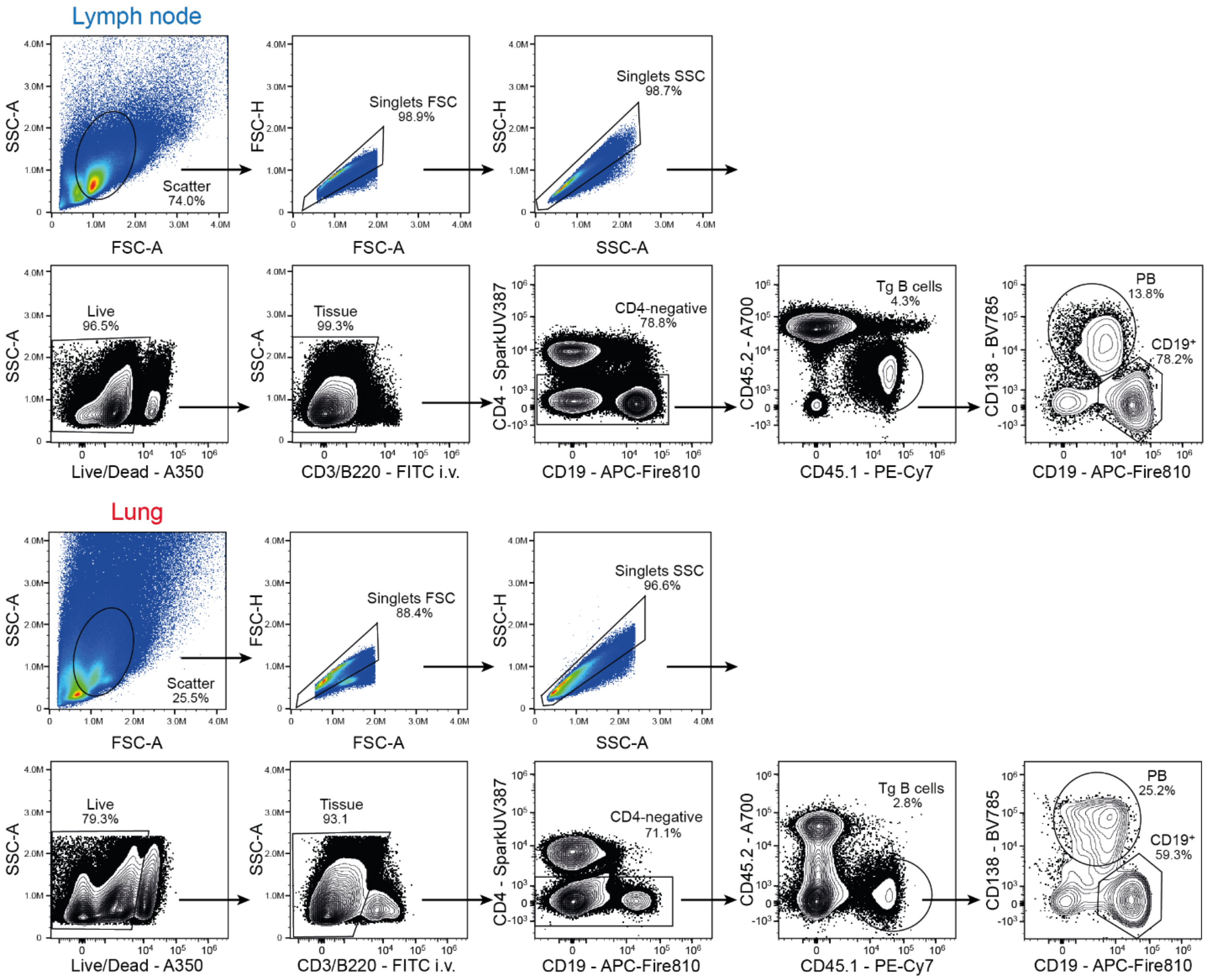

Figure S1. Gating strategy for antigen-specific CD19^{+} B cells or plasmablasts from lung-draining lymph node and lung tissue. For detection of tissue-resident cells, mice were either i.v. injected with antibodies against CD3 and B220 shortly before sacrifice, or perfused with PBS (which was tested to result in a complete removal of intravascular T and B cells).

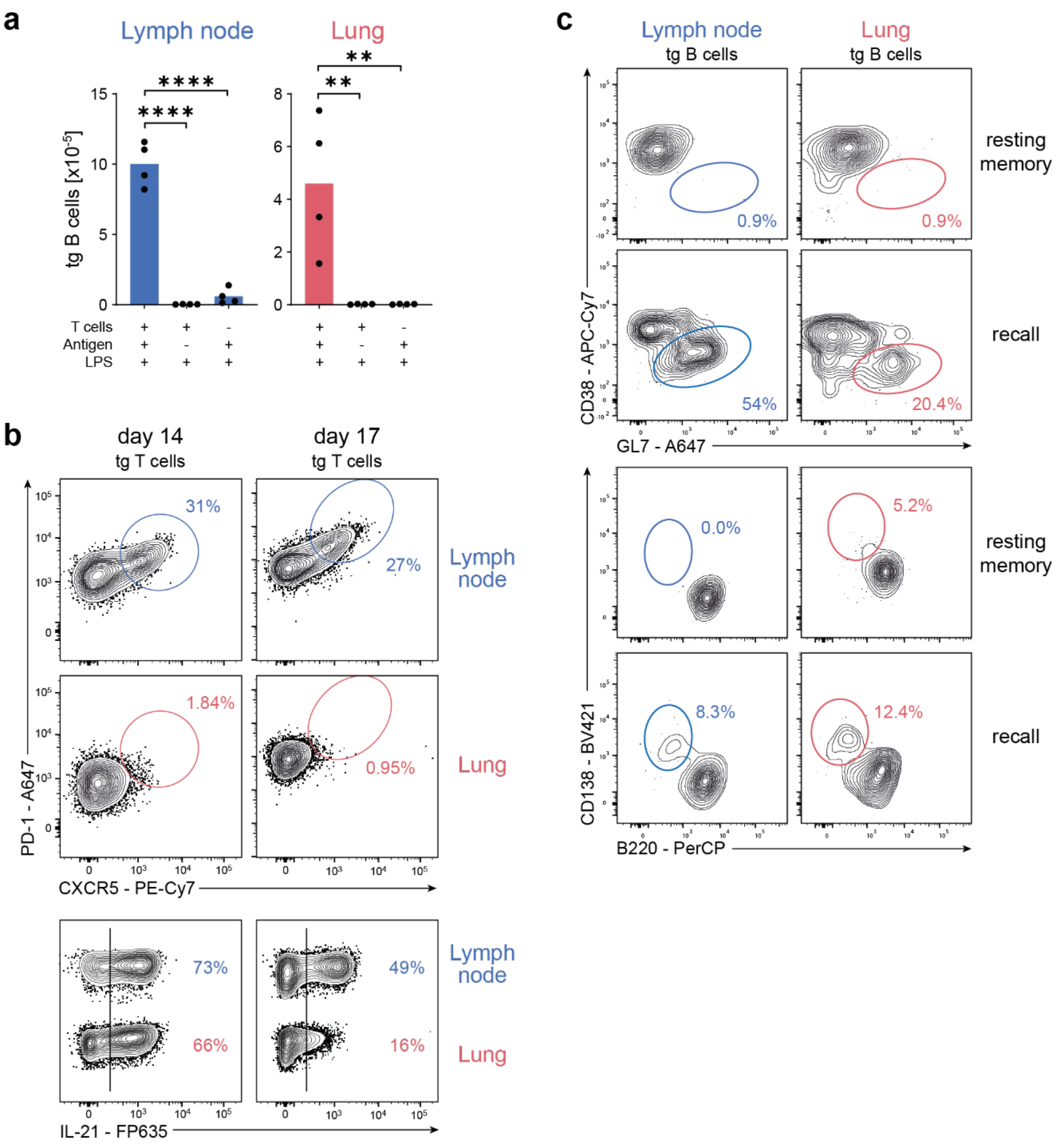

Figure S2. Lung infiltrating B cells depend on help from Tph cells and give rise to tissue-resident memory. **a,** B1-8i B cells were transferred into C57BL/6 CD28 knock-out recipients with or without co-transfer of Smarta T cells. Mice were immunized intranasally with cognate antigen and LPS or with LPS alone. Absolute numbers of transgenic B cells in lung and lung-draining lymph node were determined by flow cytometry on day 6. Dots depict four individual animals per group and bar graphs indicate the mean. **, p < 0.01; ****, p < 0.0001. **b,** Representative flow cytometry analysis of antigen-specific T cells from lung and lung-draining lymph node on day 14 (one day after the last antigen challenge) and day 17. Smarta mice crossed to an IL-21 reporter [1] were used to analyze IL-21 production directly *ex vivo*. **c,** Mice from the adoptive transfer lung inflammation model were kept without any further exposure to antigen until day 96. Mice were then rechallenged intranasally with antigen + LPS or left untreated and analyzed on day 101. Representative flow cytometry examples for both groups.

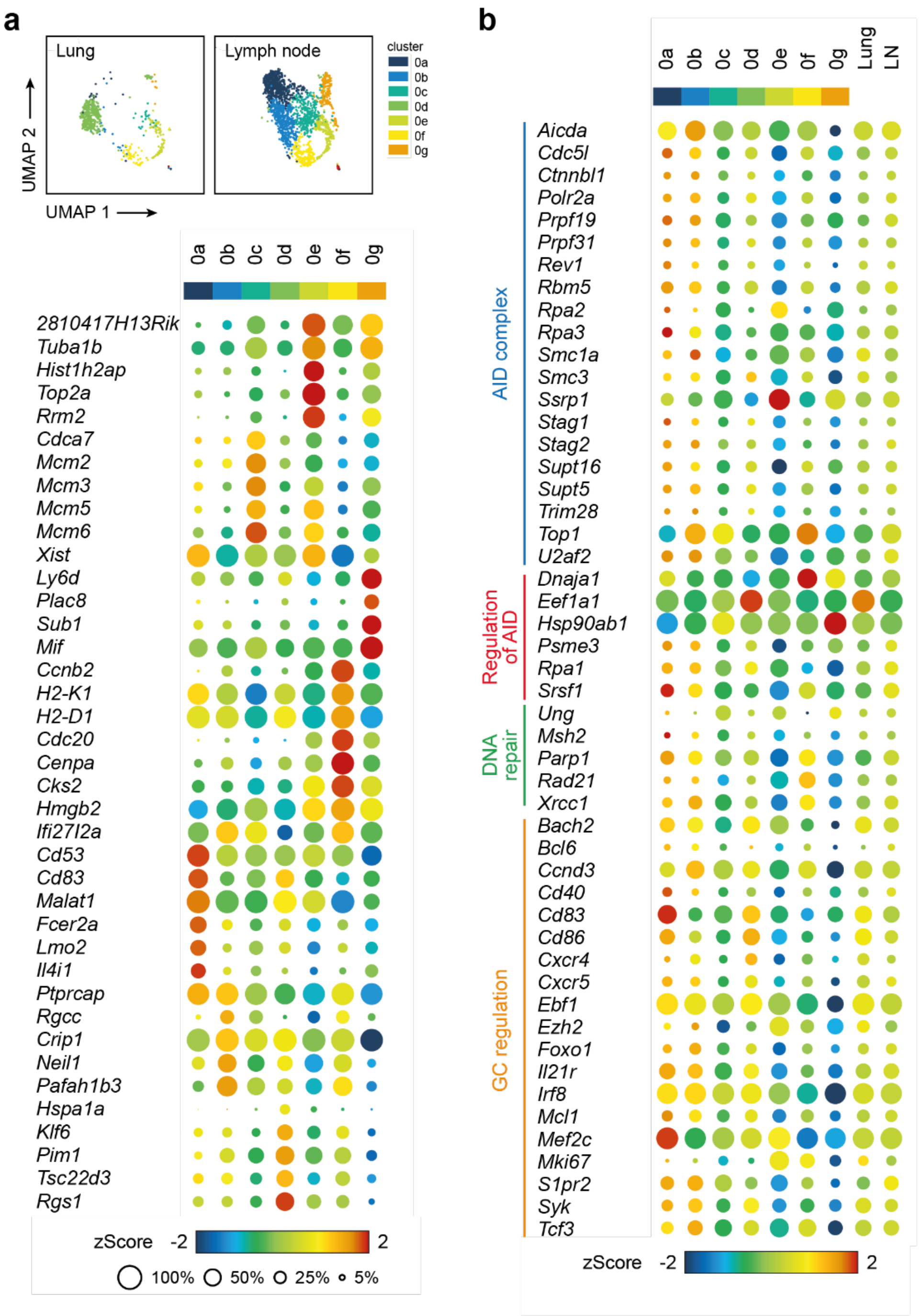

Figure S3. GC-like B cells from lung and lymph node are transcriptionally highly similar and express all relevant factors for SHM (related to Fig. 2c). Single-cell RNA-sequencing analysis of GC (- like) B cells from lung and lymph node. **a,** UMAP representation of cells and bubble plot of expression levels of the top 5 cluster-defining genes. **b,** Expression of genes relevant for SHM in the different subclusters and in all GC -like B cells from lung versus lymph node. Similar results were obtained from the second scRNA experiment with sorted GC-like B cells. The color scale shows the z-scores of the average expression of a gene within the indicated cluster. Bubble sizes correspond to the fraction of cells expressing a particular gene within the indicated cluster.

a

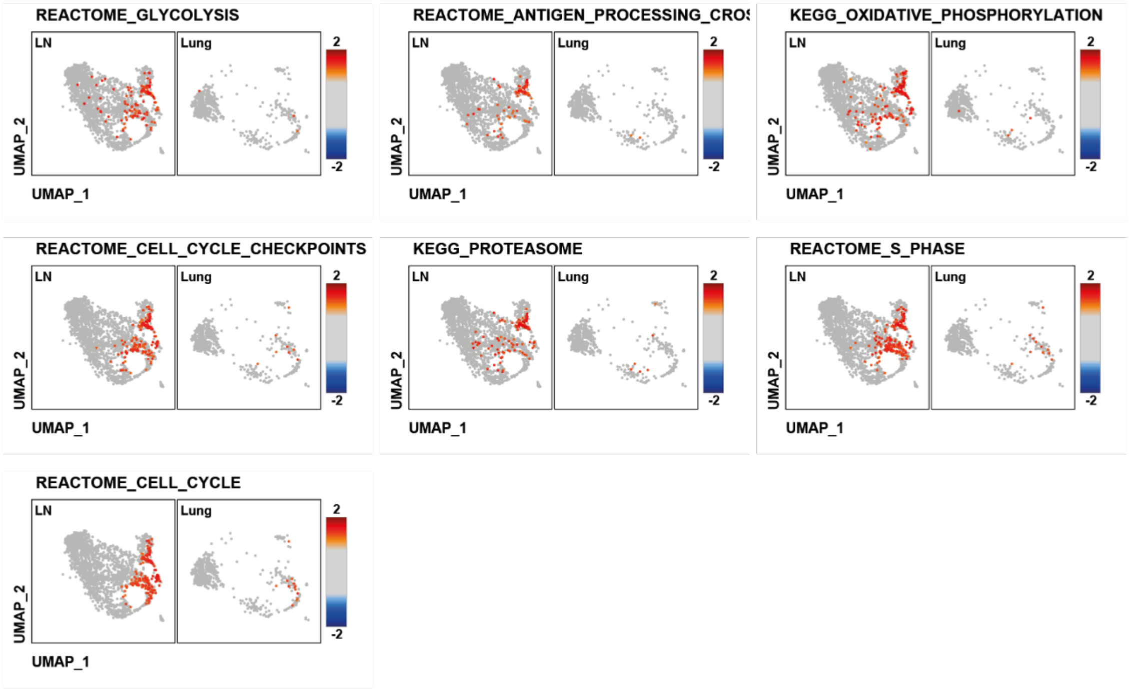

b

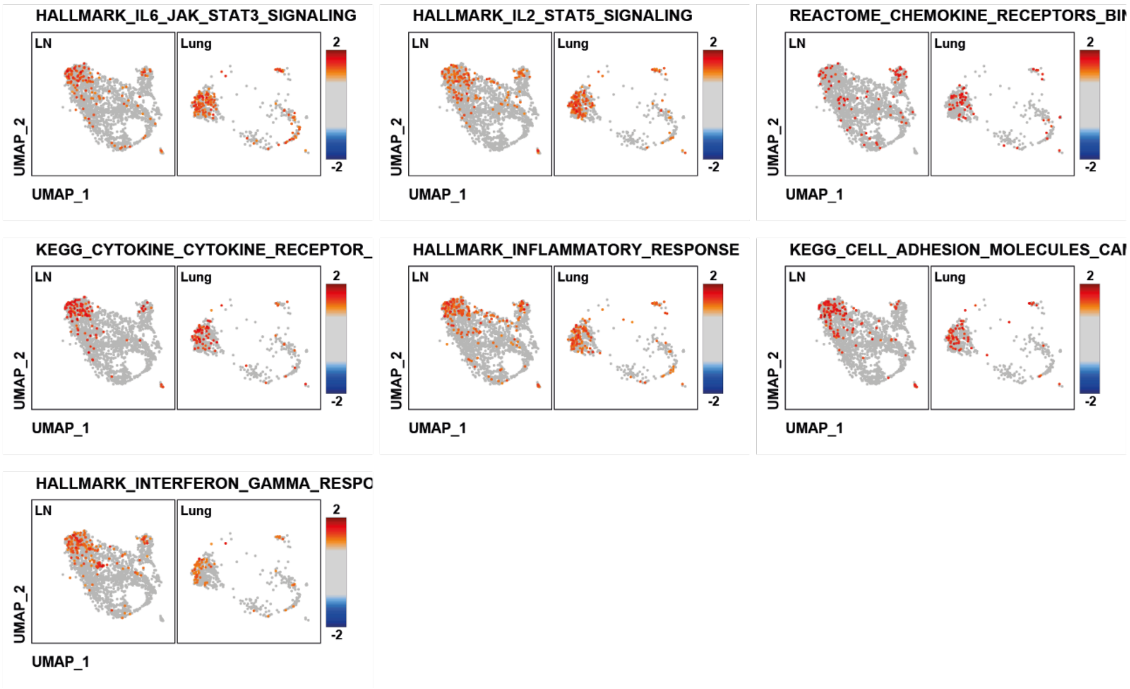

Figure S4. Pathway analysis for GC-like B cells from lung and lymph node (related to Fig. 2d). Cells expressing genes from the indicated pathways are shown in red. Similar results were obtained from the second scRNA experiment with sorted GC (-like) B cells.

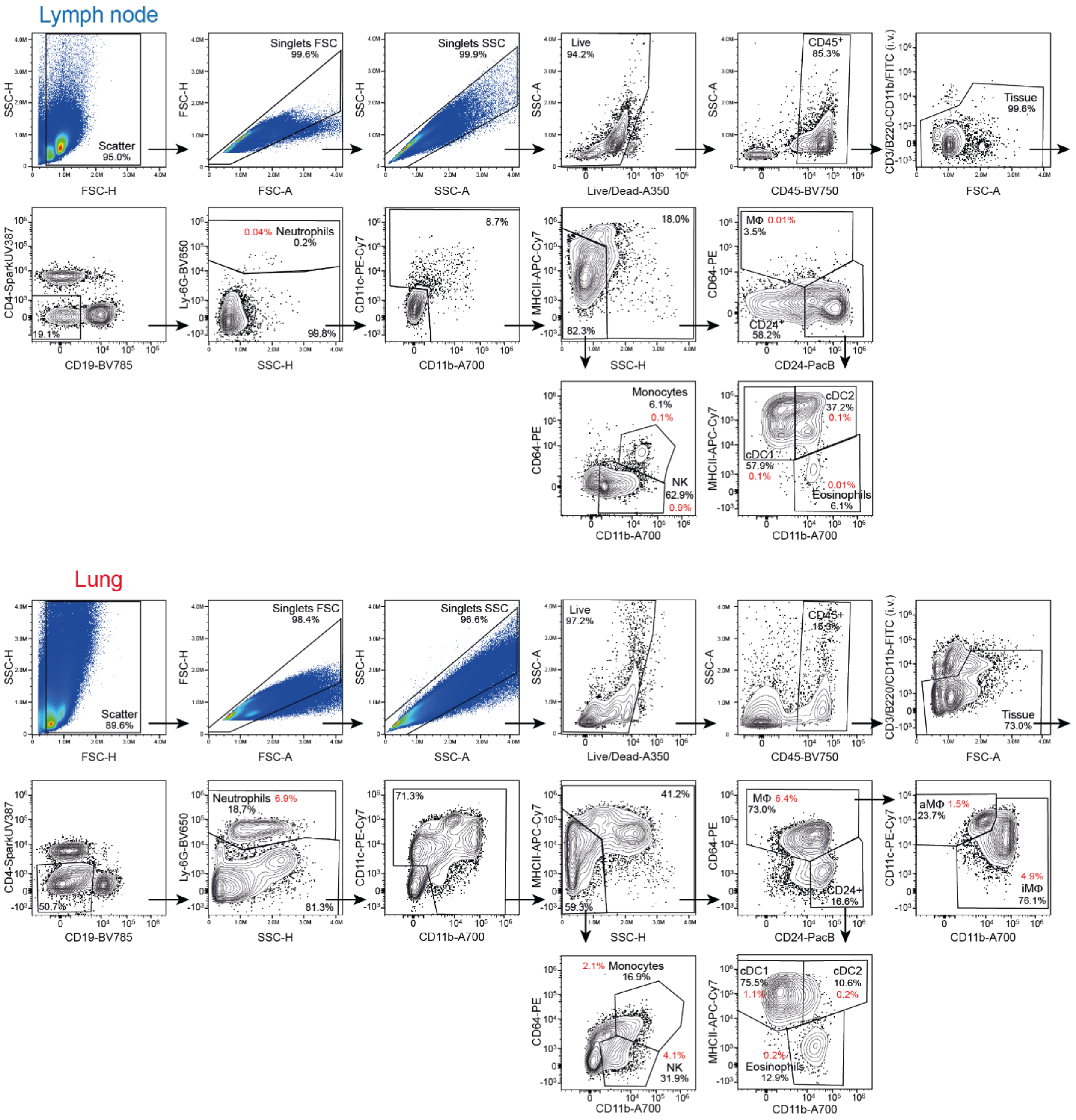

Figure S5. Gating strategy to identify different APC cell populations in lymph node and lung [2] (related to Fig. 4). Black inset numbers indicate the frequency of the parent population within the tree whereas red numbers show the population size as frequency of all $CD45^+$ cells. For all experiments with fluorescent (PE) antigen, CD64 was stained in Alexa Fluor 647.

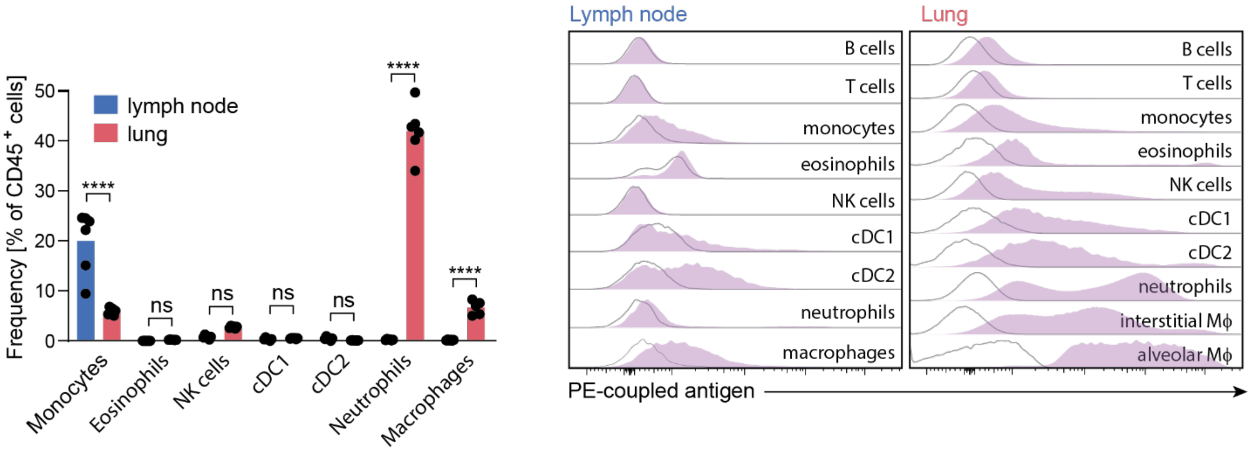

Figure S6. Lung macrophages are also a major antigen-presenting population for early B cell activation (related to Fig. 4b). Mice were immunized with antigen and LPS intranasally and analyzed 24 hours later. **a,** Comparison of the frequencies of different APC populations in lung and lymph node (see Supplementary Fig. S5 for gating). Symbols represent data from three different mice and bars the mean. ns, $p \geq 0.05$; ****, $p < 0.0001$. **b,** Antigen retention on different APC subpopulations. Histograms are concatenated from three mice either receiving fluorescent (purple filled histograms) or non-fluorescent (grey histograms) antigen. Data are from one representative experiment out of two.

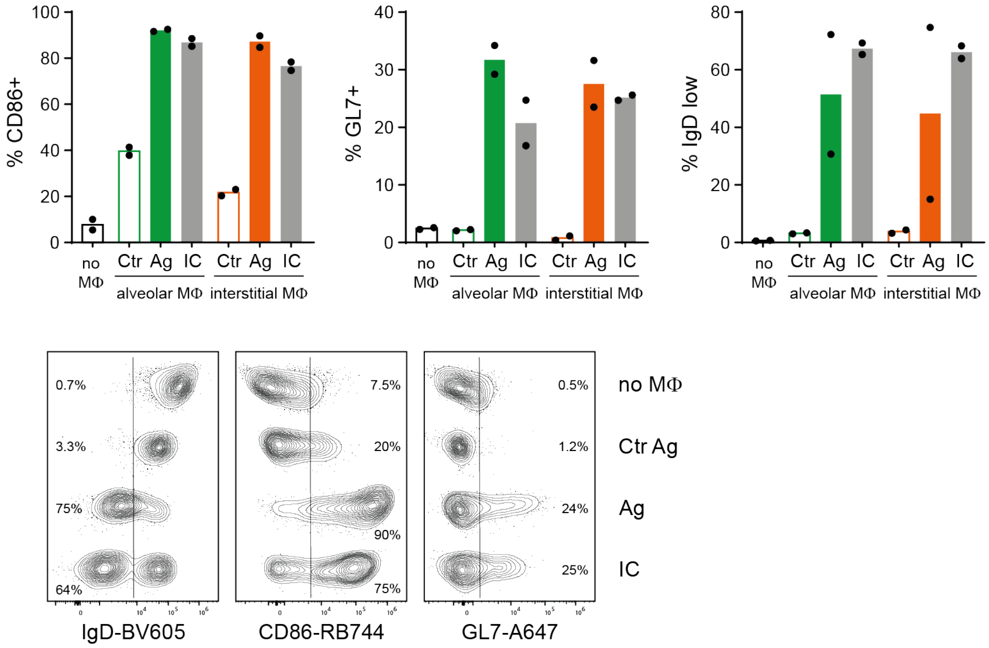

Figure S7. Phenotype of B cells after 48 hours of coculture with macrophages (related to Fig. 5a). Interstitial and alveolar macrophages were sorted (for gating refer to Supplementary Fig. S5) from the lung inflammation model on day 6. Two hours and 24 hours before sacrifice, mice received additional antigen intranasally, either Smarta peptide and NIP coupled to mouse serum albumin (Ag) or a conjugate without NIP (Ctr). Macrophages were cocultured together with naive B cells for 48 hours before the B cells were analyzed for activation markers by flow cytometry. As a positive control, immune-complexed antigen (IC; antigen complexed with anti-NP IgM) was directly added to the cultures. The bar graphs represent the means of duplicate cultures (dots) from a single experiment, and representative flow plots from the coculture with interstitial macrophages are below.

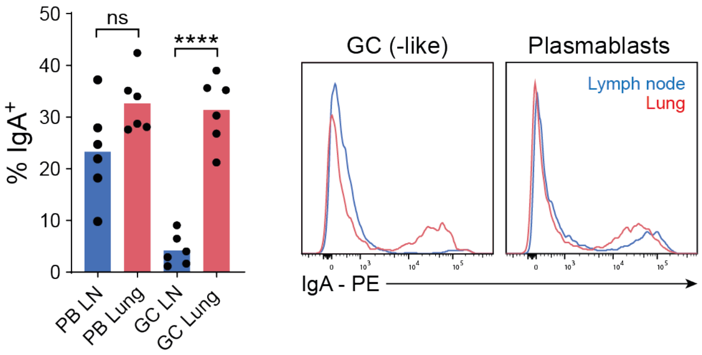

Figure S8. IgA⁺ GC-like B cells are exclusively found in the lung but not in the lung-draining lymph node (related to Fig. 7b). Antigen-specific B cells from lung and lung-draining lymph node (day 17) gated either for a GC (-like) or plasmablast phenotype (see Supplementary Fig. S1) were analyzed for expression of IgA. Representative experiment out of two with five mice. Dots depict individual animals and bar graphs indicate the mean. ns, $p \geq 0.05$; ****, $p < 0.0001$.

a

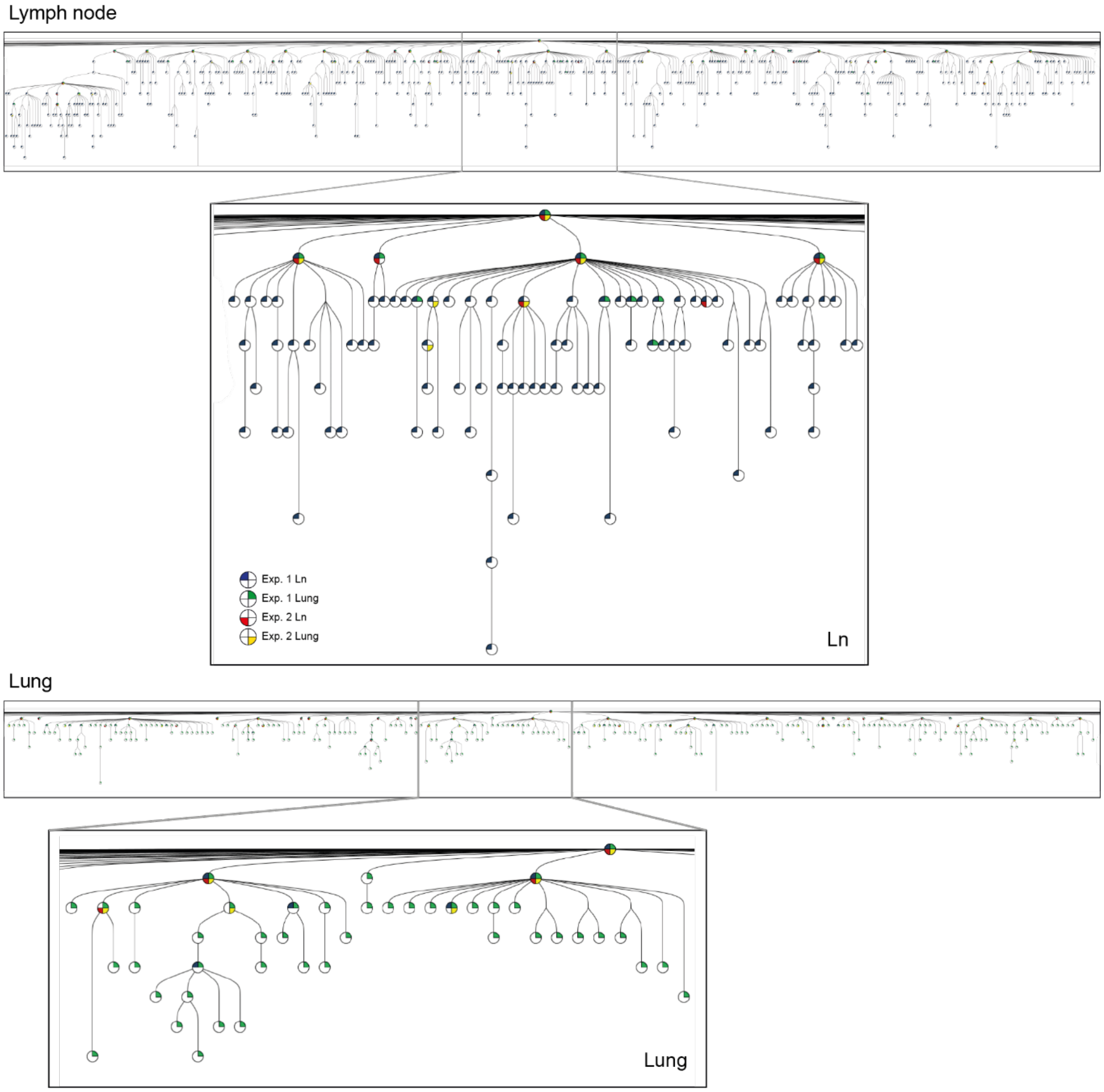

b

Comparison of experimental and random overlap					
		Ln Exp. 1	Lung Exp. 1	Ln Exp. 2	Lung Exp. 2
experiment	Ln Exp. 1	100%	0.96%	0.41%	0.58%
	Lung Exp. 1	1.55%	100%	0.56%	1.86%
	Ln Exp. 2	0.43%	0.36%	100%	0.29%
	Lung Exp. 2	1.85%	3.70%	0.90%	100%
random simulation	Ln Exp. 1	100%	4.06%	5.28%	2.27%
	Lung Exp. 1	6.25%	100%	5.85%	2.61%
	Ln Exp. 2	5.78%	4.16%	100%	2.34%
	Lung Exp. 2	7.15%	5.34%	6.71%	100%
p-value lower than expected	Ln Exp. 1	ns	< 0.001	< 0.001	< 0.001
	Lung Exp. 1	< 0.001	ns	< 0.001	< 0.001
	Ln Exp. 2	< 0.001	< 0.001	ns	< 0.001
	Lung Exp. 2	< 0.001	< 0.001	< 0.001	ns

Figure S9. Clonal tree analysis of B cells from lymph node and lung (bulk RNA sequencing; related to Fig. 7e). In two independent experiments, GC-like B cells were sorted from antigen-specific B cells in lung and lung-draining lymph node (pooled cells from 10 animals). The heavy chain BCR sequence was analyzed using a bulk sequencing protocol [3]. Lineage trees from both organs were computed with GLaMST [4]. **a,** The top panels for lymph node and lung show part of this tree (from Exp. 1). The enlargement shows the germline sequence on top with some exemplary trees. Color codes indicate whether the sequence is unique to lymph node or lung (blue or green only) or whether the identical sequence was found in both organs (blue and green). If the same sequence was also found in the second independent experiment (combination with red or yellow), this indicates that the overlap is most likely random. As anticipated, the germline sequence and numerous sequences with only one mutation were detected in both the lung and lymph node. However, as identical sequences were also detected in clones from an independent experiment, this overlap seems to be rather coincidental. Conversely, the majority of clones with more than one mutation were unique to either the lung or lymph node. **b,** Experimentally observed overlap of clones between organs and experiments and results from a random simulation. Cells were shuffled 1,000 times between samples. Only clones with more than one mutation were included in this analysis. The p-values indicate whether the experimentally observed overlap is significantly lower than the expected overlap.

Overlap of randomly selected Ln clones to lung cells			
	Ln 1	Ln 2	Lung
Ln 1	100%	5.48%	1.56%
Ln 2	5.47%	100%	1.56%
Lung	1.10%	1.10%	100%

Figure S10. Exclusion of over- or undersampling effects (related to Fig. 7e and f). To exclude sampling effects as a reason for the low clonal overlap between lymph node and lung, 600 randomly selected cells from all lymph node GC B cells with more than one mutation were divided into two pools and compared to 300 lung GC-like clones. The random sampling was repeated 10,000 times to ensure robust results. The overlap between two lymph node clone pools is significantly higher than the overlap of a lymph node to lung cells of similar size ($p = 0.0018$).

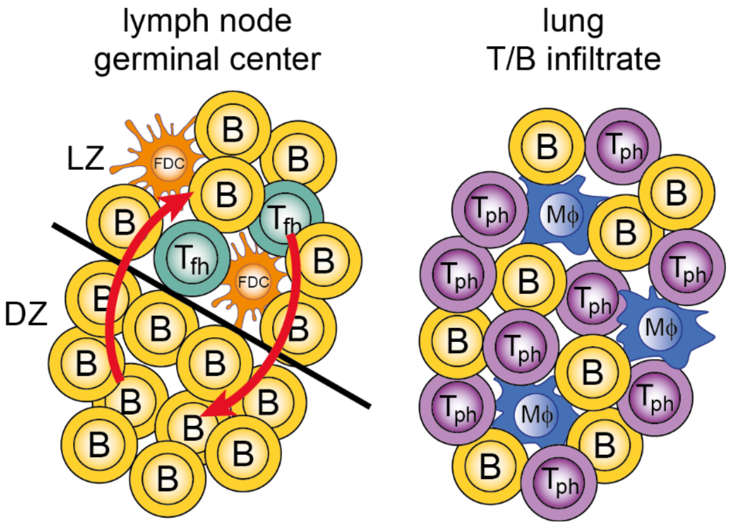

Figure S11. B cell selection in the lung takes places in a less controlled environment. SLO provide a complex microenvironment to optimize selection and affinity maturation of antigen-specific B cells. SLO are segregated into T cell and B cell zones, and only a limited number of antigen-specific T cells, which upregulate CXCR5 during their activation in the T cell zone, are able to enter the B cell zone. GC B cells typically outnumber Tfh cells by a factor of ten [5], ensuring that T cell help for antigen-specific B cells is limited. As a result, only B cells with the highest affinity BCR capture and present enough antigen to receive efficient help from Tfh cells [6, 7]. The GC is further subdivided into a light zone (LZ), where B cells see their antigen on the FDC and receive T cell help, and a dark zone (DZ), where they proliferate and hypermutate their BCR in the absence of any selection [8]. B cell shuttling between dark and light zone is considered as an additional safeguard mechanism against selection of autoreactive B cells. B cells that lose their original antigen specificity due to hypermutation in the DZ do no longer present the original peptides when they re-enter the LZ and thus do not receive any T cell help [9]. In contrast, lung GC-like B cells have constant access to antigen presented by macrophages (Mϕ) and help from Tph cells which are also more numerous compared to Tfh cells in the lymph node.

Table S1: Antibodies for flow cytometry

Specificity	Clone/reagent	Conjugate	Source
Bcl-6	K112-91	PE	BD Biosciences
Biotin	Streptavidin	PerCP	BioLegend
Biotin	Streptavidin	PE-Fire 700	BioLegend
CD3	KT3	FITC	Own conjugate
CD3	KT3	PE	Own conjugate
CD4	GK1.5	FITC	Own conjugate
CD4	GK1.5	Briliant Violet 750	BioLegend
CD4	GK1.5	Spark UV 387	BioLegend
CD4	GK1.5	Pacific Orange	Own conjugate
CD4	RM4-5	Brilliant Violet 711	BioLegend
CD8a	53-6.7	FITC	Own conjugate
CD8a	53-6.7	Pacific Orange	Own conjugate
CD8a	53-6.7	Spark UV 387	BioLegend
CD8a	53-6.7	Brilliant Violet 711	BioLegend
CD11b	5C6	Alexa Fluor 700	Own conjugate
CD11b	5C6	FITC	Own conjugate
CD11c	N418	PE-Cy7	Own conjugate
CD19	1D3	FITC	Own conjugate
CD19	6D5	APC-Fire 810	BioLegend
CD19	6D5	Brilliant Violet 785	BioLegend
CD24	M1/69.16.11.HL	Pacific Blue	Own conjugate
CD38	90	APC-Cy7	BioLegend
CD38	90	Pacific Blue	BioLegend
CD45	30-F11	Brilliant Violet 750	BioLegend
CD45.1	A20	PerCP	BioLegend
CD45.1	A20	PE-Cy7	BioLegend
CD45.1	A20	Biotin	BioLegend
CD45.1	REA1179	PerCP-Vio 700	Miltenyi
CD45.2	104	Alexa Fluor 700	BioLegend
CD45.2	104	Brilliant Violet 750	BioLegend
CD45R (B220)	RA3-6B2	FITC	Own conjugate
CD45R (B220)	RA3-6B2	PE	Own conjugate
CD45R (B220)	RA3-6B2	PerCP	BioLegend
CD62L	Mel-14	FITC	Own conjugate
CD62L	Mel-14	PE-Fire810	BioLegend
CD64	X54-5/7.1	Alexa Fluor 647	BioLegend
CD64	X54-5/7.1	PE	BioLegend
CD69	H1.2F3	Brilliant UV 737	BD Biosciences
CD69	H1.2F3	PE-Cy5	BioLegend
CD86	GL1	RealBlue 744	BD Biosciences
CD90.1	OX-7	Alexa Fluor 700	Own conjugate
CD90.1	OX-7	Brilliant Violet 711	BioLegend
CD90.1	OX-7	Pacific Blue	Own conjugate
CD138	281-2	Brilliant Violet 785	BioLegend
CD138	281-2	PE	BioLegend
CXCR5	L138D7	PE-Dazzle 594	BioLegend
CXCR5	L138D7	Biotin	BioLegend
GL7	GL7	Alexa Fluor 647	Own conjugate
GL7	GL7	PE-Cy7	BioLegend
I-A/I-E	M5/114.15.2	APC-Cy7	Own conjugate

Table S1 (continued)

Specificity	Clone/reagent	Conjugate	Source
IgA	mA-6E1	PE	Invitrogen
IgD	11-26c	Brilliant Violet 605	BioLegend
Ki-67	SolA15	eFluor 450	eBioscience
Ki-67	B56	BUV395	BD Biosciences
Ly-6C	HK1.4	PerCP	BioLegend
Ly-6G	1A8	Brilliant Violet 650	BD Biosciences
PD-1	J43	APC	eBioscience
PD-1	J43	PE	eBioscience

Table S2: Antibodies for immunohistology

Specificity	Clone/reagent	Conjugate	Supplier
CD21/35	7G6	Alexa Fluor 546	Own conjugate
CD21/35	7G6	Alexa Fluor 647	Own conjugate
CD45.1	A20	FITC	eBioscience
CD45.1	REA1179	Biotin	Miltenyi
CD90.1	OX-7	Digoxigenin	Own conjugate
CD90.1	REA838	Biotin	Miltenyi
Digoxigenin	Sheep polyclonal	POD	Merck
ER-TR7	ER-TR7	Alexa Fluor 647	Santa Cruz
F4/80	F4/80	Digoxigenin	Own conjugate
FITC	Sheep polyclonal	POD	Merck
IgD	11-26c	Alexa Fluor 594	Own conjugate
Ly-6G	1A8	FITC	BD Biosciences
Ki-67	B56	FITC	eBioscience
Rabbit IgG	Goat polyclonal	Alexa Fluor 555	Invitrogen
Goat IgG	Donkey polyclonal	POD	Santa Cruz
Human IgG	Goat polyclonal	Alexa Fluor plus 488	Invitrogen
Human IgG	Donkey polyclonal	Dylight650	Invitrogen

*POD: horseradish peroxidase

Table S3: TaqMan Gene Expression Assays

Hprt	*Mm01545399_m1*
Il6	*Mm00446190_m1*
Il10	*Mm01288386_m1*
Il12b	*Mm01288989_m1*
Tgfb1	*Mm01178820_m1*
Tnfsf13b (BAFF)	*Mm00446345_m1*

**Table S4: Parameter values for the *in silico* model**				
parameter	symbol	value	unit	source
initial No of clones	N_{cl}	50.0		(1)
GC capacity	C	1,000.0		(2)
growth phase duration	T_g	5.0	d	(2)
competition phase duration	T_c	9.0	d	(2)
Onset time of growth decay in the lung	T_{dec}	8.0	d	
initial affinity	w_0	0.1		(1)
mutation rate	D	0.01		(2)
base-level death rate	δ_0	1.0	1/d	(1)
base-level growth rate	λ_0	1.2	div./d	
growth rate in growth phase		$2*\lambda_0$		(2)
growth rate in lymph node (DZ)		$3*\lambda_0$		(3)
max affinity-induced fold-increase in λ	g_λ	3.0		
max affinity-induced fold-decrease in δ	g_δ	3.0		
transition rate LZ->DZ	k_{LZDZ}	0.04	hr^{-1}	(4)
transition rate DZ->LZ	k_{DZLZ}	0.5	hr^{-1}	(4)
Decay coefficient for growth rate in lung	κ	0.53	hr^{-1}	Fig. 6
Growth-rate modification factor lymph node	α_{LN}	2		

(1) original parameter values from [10]

(2) parameters from [10] modified to realize shorter growth and competition phases in line with experimental data

(3) Base-level growth rate is higher in DZ compared to SLO scenario; see text.

(4) adopted from [11]

REFERENCES

1. Shulman Z, Gitlin AD, Weinstein JS, Lainez B, Esplugues E, Flavell RA, et al. Dynamic signaling by T follicular helper cells during germinal center B cell selection. Science. 2014;345:1058-62.
2. Yu YR, O'Koren EG, Hotten DF, Kan MJ, Kopin D, Nelson ER, et al. A Protocol for the Comprehensive Flow Cytometric Analysis of Immune Cells in Normal and Inflamed Murine Non-Lymphoid Tissues. PLoS One. 2016;11:e0150606.
3. Turchaninova MA, Davydov A, Britanova OV, Shugay M, Bikos V, Egorov ES, et al. High-quality full-length immunoglobulin profiling with unique molecular barcoding. Nat Protoc. 2016;11:1599-616.
4. Yang X, Tipton CM, Woodruff MC, Zhou E, Lee FE, Sanz I, et al. GLaMST: grow lineages along minimum spanning tree for b cell receptor sequencing data. BMC Genomics. 2020;21:583.
5. Wittenbrink N, Klein A, Weiser AA, Schuchhardt J, Or-Guil M. Is there a typical germinal center? A large-scale immunohistological study on the cellular composition of germinal centers during the hapten-carrier-driven primary immune response in mice. J Immunol. 2011;187:6185-96.
6. Victora GD, Schwickert TA, Fooksman DR, Kamphorst AO, Meyer-Hermann M, Dustin ML, et al. Germinal center dynamics revealed by multiphoton microscopy with a photoactivatable fluorescent reporter. Cell. 2010;143:592-605.
7. Gitlin AD, Shulman Z, Nussenzweig MC. Clonal selection in the germinal centre by regulated proliferation and hypermutation. Nature. 2014;509:637-40.
8. Victora GD, Nussenzweig MC. Germinal Centers. Annu Rev Immunol. 2022;40:413-42.
9. Bannard O, McGowan SJ, Ersching J, Ishido S, Victora GD, Shin JS, et al. Ubiquitin-mediated fluctuations in MHC class II facilitate efficient germinal center B cell responses. J Exp Med. 2016;213:993-1009.
10. Amitai A, Mesin L, Victora GD, Kardar M, Chakraborty AK. A Population Dynamics Model for Clonal Diversity in a Germinal Center. Front Microbiol. 2017;8:1693.
11. Meyer-Hermann M, Mohr E, Pelletier N, Zhang Y, Victora GD, Toellner KM. A theory of germinal center B cell selection, division, and exit. Cell Rep. 2012;2:162-74.
